# Tumorigenic circulating tumor cells from xenograft mouse models of non-metastatic NSCLC patients reveal distinct single cell heterogeneity and drug responses

**DOI:** 10.1186/s12943-022-01553-5

**Published:** 2022-03-12

**Authors:** Kanve N. Suvilesh, Yulia I. Nussbaum, Vijay Radhakrishnan, Yariswamy Manjunath, Diego M. Avella, Kevin F. Staveley-O’Carroll, Eric T. Kimchi, Aadel A. Chaudhuri, Chi-Ren Shyu, Guangfu Li, Klaus Pantel, Wesley C. Warren, Jonathan B. Mitchem, Jussuf T. Kaifi

**Affiliations:** 1 Department of Surgery, Ellis Fischel Cancer Center, University of Missouri, One Hospital Drive, Columbia, MO 65212 USA; 2 Institute for Data Science and Informatics, University of Missouri, Columbia, MO USA; 3 Harry S. Truman Memorial Veterans’ Hospital, Columbia, MO USA; 4 Siteman Cancer Center, Washington University School of Medicine, St. Louis, MO USA; 5 Department of Radiation Oncology, Washington University School of Medicine, St. Louis, MO USA; 6 Institute for Tumor Biology, University of Hamburg, Hamburg, Germany; 7 Bond Life Sciences Center, University of Missouri, Columbia, MO USA

## Abstract

**Background:**

Circulating tumor cells (CTCs) are liquid biopsies that represent micrometastatic disease and may offer unique insights into future recurrences in non-small cell lung cancer (NSCLC). Due to CTC rarity and limited stability, no stable CTC-derived xenograft (CDX) models have ever been generated from non-metastatic NSCLC patients directly. Alternative strategies are needed to molecularly characterize CTCs and means of potential future metastases in this potentially curable patient group.

**Methods:**

Surgically resected NSCLC primary tumor tissues from non-metastatic patients were implanted subcutaneously in immunodeficient mice to establish primary tumor patient-derived xenograft (ptPDX) models. CTCs were isolated as liquid biopsies from the blood of ptPDX mice and re-implanted subcutaneously into naïve immunodeficient mice to generate liquid biopsy CTC-derived xenograft (CDX) tumor models. Single cell RNA sequencing was performed and validated in an external dataset of non-xenografted human NSCLC primary tumor and metastases tissues. Drug response testing in CDX models was performed with standard of care chemotherapy (carboplatin/paclitaxel). Blockade of MYC, which has a known role in drug resistance, was performed with a MYC/MAX dimerization inhibitor (10058-F4).

**Results:**

Out of ten ptPDX, two (20%) stable liquid biopsy CDX mouse models were generated. Single cell RNA sequencing analysis revealed an additional regenerative alveolar epithelial type II (AT2)-like cell population in CDX tumors that was also identified in non-xenografted NSCLC patients’ metastases tissues. Drug testing using these CDX models revealed different treatment responses to carboplatin/paclitaxel. MYC target genes and c-MYC protein were upregulated in the chemoresistant CDX model, while MYC/MAX dimerization blocking could overcome chemoresistance to carboplatin/paclitaxel.

**Conclusions:**

To overcome the lack of liquid biopsy CDX models from non-metastatic NSCLC patients, CDX models can be generated with CTCs from ptPDX models that were originally established from patients’ primary tumors. Single cell analyses can identify distinct drug responses and cell heterogeneities in CDX tumors that can be validated in NSCLC metastases tissues. CDX models deserve further development and study to discover personalized strategies against micrometastases in non-metastatic NSCLC patients.

**Supplementary Information:**

The online version contains supplementary material available at 10.1186/s12943-022-01553-5.

## Main text

Non-small cell lung cancer (NSCLC) is a devastating disease with high mortality. Even patients with early-stage disease amenable to resection have a 50% mortality rate [1]. Cancer recurrence is directly linked to the presence of radiographically undetectable micrometastatic disease, such as circulating tumor cells (CTCs) in the blood [2, 3]. CTCs that shed into the bloodstream serve as liquid biopsies and can be tumorigenic [4, 5]. CTC-derived xenograft (CDX) models therefore represent an opportunity to study the evolution of metastasis; however, they have been exclusively established from patients that already have metastatic disease [5]. Due to the rarity of CTCs in non-metastatic NSCLC patients, CDX models in this critical patient group are lacking. To overcome the limitations of CDX model development from non-metastatic NSCLC patients, in this study two stable CDX mouse models were generated using primary tumor patient-derived xenograft (ptPDX)-derived CTCs. Single cell analysis of ptPDX and CDX tumors revealed the existence of an additional, regenerative alveolar epithelial cell type II (AT2)-like population in CDX tumors that was also identified in non-xenografted NSCLC metastases. Additionally, in one CDX model chemoresistance could be overcome by inhibition of MYC, a known contributor to drug resistance [6]. Further study of CDX models might be critical to design therapies targeting micrometastatic disease that prevents recurrences in non-metastatic NSCLC patients [7–9].

## Results and discussion

### Development of NSCLC CDX mouse models with ptPDX-derived CTCs

**Fig. 1:**
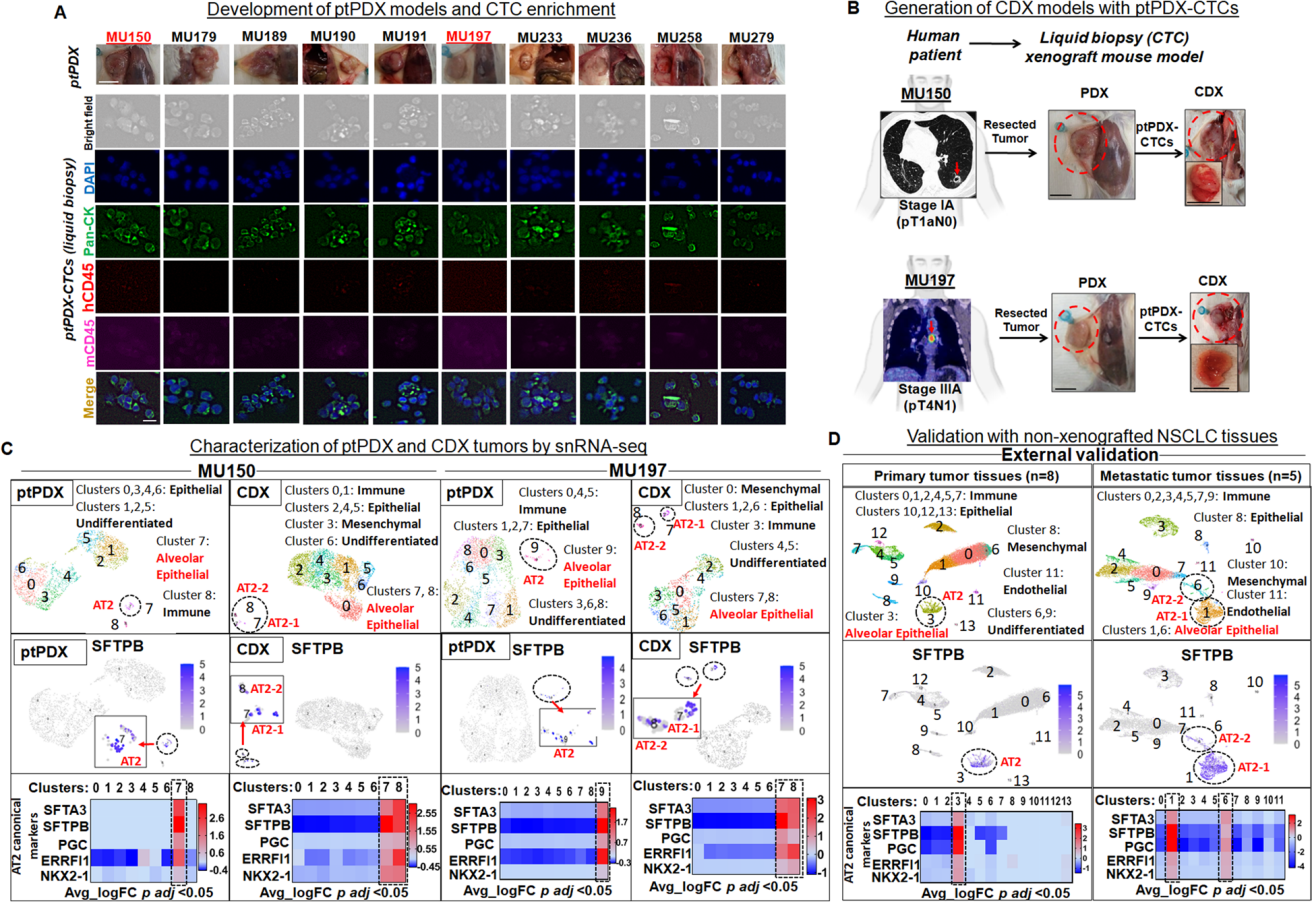
Liquid biopsy CDX mouse model generation and single cell sequencing analyses. **A** Upper panels: Surgically resected NSCLC primary tumor fragments were implanted into NSG mice to develop ptPDXs. Images of ptPDX tumor-bearing mice at euthanasia (with patient IDs) showing the subcutaneous tumors; Scale bar, 1 cm. Lower panels: Blood was collected from ptPDX mice at the time of euthanasia and CTCs enriched. Human CTCs were confirmed by immunostaining using anti-human Pan-CK (FITC), hCD45 (DSRed) and mCD45 (Cy5) with DAPI for nuclei identification; Scale bar, 20 μm. **B** Development of CDX mouse models from non-metastatic NSCLC patients: Representative images of the two NSCLC patients’ CT (MU150) and PET/CT (MU197) imaging showing primary tumors (red arrows) that led to CDX model development following s.c. reimplantation of ptPDX-CTCs into naïve NSG mice. Images of subcutaneous CDX tumors (dotted circles) are shown. Insets: Excised tumors. Scale bar, 1 cm. **C** Single nuclear (sn)RNA-seq transcriptome landscape of MU150 ptPDX, MU150 CDX, MU197 ptPDX and MU197 CDX visualized by uniform manifold approximation and projection (UMAP) (upper panels). Middle panels: Feature plots of SFTPB with dotted circles highlighting the AT2-like clusters (zoomed insets). Lower panels: Heat maps of AT2 cell type canonical markers using differentially expressed genes (DEGs) of all clusters of all samples confirming the existence of additional AT2-like cluster in CDX tumors. Dotted lines highlight AT2 clusters. Cell type canonical markers are provided in supplementary file 2. **D** An external single cell sequencing data set of non-xenografted human NSCLC tumor tissues (eight primary tumors and five metastases) [10] was analyzed to validate single cell sequencing findings observed in ptPDX/CDX models. Upper panels: Single cell transcriptome landscape of human primary tumor (left) and metastases (right) tissues are visualized by UMAP. Middle panels: Feature plots of SFTPB with dotted circles highlighting the AT2-like clusters in primary tumor (left) and metastases (right) tissues. Lower panels: Heat maps of AT2 cell type canonical markers using differentially expressed genes (DEGs) of all clusters of primary tumors (left) and metastases (right) tissues. Dotted lines highlight AT2 clusters

Primary tumor tissues from non-metastatic NSCLC patients were collected at the time of lung resection (Table S1). Ten ptPDX models were generated by growing primary tumor fragments following subcutaneous (s.c.) implantation in immunodeficient NOD scid gamma (NSG) mice (Fig. 1A; upper panels). CTCs from ptPDX mice blood were isolated, enriched, and immunostained with traditional CTC identification criteria (Fig. 1A; lower panels). CTCs from ptPDX phenotypically matched CTCs isolated from patients (Fig. S1). CTCs isolated from ptPDX were then injected subcutaneously in naïve NSG mice to establish CDX. ptPDX-derived CTCs developed into stable CDX tumors in two out of ten (20%) patients (MU150, MU197) (Fig. 1B; Table S1). High numbers of CTC clusters present in the ptPDX mice may be a driving factor for successful CDX development (Table S2). CTC clusters have enhanced metastatic potential [11], and in PDX models CTC clusters correlated with metastatic development [12]. Pathology-reported biomarker expressions in resected patient primary tumors were conserved in matched ptPDX and CDX tumors (Fig. S2 A/B).

Due to the rarity and instability of micrometastatic CTCs, stable CTC expansion models from non-metastatic NSCLC patients are lacking. Although xenograft models represent a clonal cell selection, ptPDX-derived CDX liquid biopsy models can still be a valuable tool to molecularly study and predict the risk of future recurrences and metastases after curative resection of localized NSCLCs in individual patients.

### Single cell analysis revealed an additional regenerative AT2-like population in CDX tumors and human patients’ NSCLC metastases

To generate global atlases of the transcriptomic landscape, ptPDX and CDX tumors were profiled by single nuclear RNA-sequencing (snRNA-seq) (Figs. 1C, S3, S4). Nine cell type clusters across all samples were visualized, except for MU197 ptPDX that had ten clusters (Fig. 1C; upper panels). Cell types were identified using differentially expressed genes (DEGs) expressing canonical, cell-specific markers (Fig. S5). As expected in xenograft tumors, most of the cell clusters were of epithelial origin. CDX tumors had two AT2-like cell populations, in contrast to ptPDX tumors that had just one. Feature plots of AT2-specific marker surfactant protein B (SFTPB) confirmed the presence of these populations (Fig. 1C; middle panels). Heatmaps of AT2 canonical marker expression in DEGs of all clusters also showed presence of an additional AT2-like cluster in CDX tumors (Fig. 1C; lower panels).

To validate our findings, we utilized an external single cell sequencing data set consisting of eight NSCLC primary tumors and five metastases [10]. This analysis of non-xenografted tumors showed 14 clusters in the primary tumor and 12 clusters in metastatic tissues (Fig. 1D; upper panels) that were annotated, as above (Fig. S6). Similar to the observation in CDX tumors, an additional AT2-like cluster was present in metastases, but not in primary tumors. Feature plots of the AT2-specific marker SFTPB verified an additional AT2-like population in metastatic NSCLC tissues (Fig. 1D; middle panels). Additionally, heatmaps showing AT2 canonical marker expression in DEGs of all clusters of primary and metastatic patient tumor tissue samples recapitulated the findings observed in ptPDX and CDX tumors, respectively (Fig. 1D; lower panels).

Single cell sequencing demonstrated the existence of an additional AT2-like cell population in metastatic CDX and, importantly, also in non-xenografted NSCLC metastases tissues. It is well reported that AT2 cells, apart from their regenerative stem cell capacity in non-cancerous lung, are found in lung cancers and have been linked to tumor initiation demonstrated in preclinical models [13, 14]. A recent single cell sequencing analysis on advanced-stage NSCLC tissues identified an AT2-like population expressing cell proliferation and migration genes [15]. These findings support our results suggesting that AT2-like cells may have a role in metastasis development.

### Gene expression similarities and differences within and across patient-matched PDX/CDX models correlate with aggressive tumor growth

To explore the similarities and differences of the patient-matched PDX/CDX models, we aggregated snRNA-seq of PDX and CDX samples. MU150 PDX was strikingly different than MU150 CDX, as determined by DEGs following aggregation (Fig. S7 A). Gene set enrichment analysis (GSEA) [16] and pathway enrichment analysis [17] performed using top DEGs showed that cell adhesion/migration genes were commonly upregulated in MU150 PDX/CDX, whereas MU150 CDX had higher enrichment of ERBB signaling and Rho GTPase pathway genes (Fig. S7 B). Unlike MU150 PDX/CDX, MU197 PDX DEGs were not very different from MU197 CDX (Fig. S7 D). However, MU197 CDX showed enrichment of cell adhesion/migration ERBB signaling and Rho GTPase pathway genes, similar to MU150 CDX (Fig. S7 E). It is well reported that cell adhesion/migration, ERBB signaling, and Rho GTPase pathway gene upregulation leads to increased aggressiveness in cancer, including NSCLC [18–20]. MU150/197 CDXs were more aggressive in tumor growth compared to their parental PDXs (Fig. S7 C/F). To explore the similarities and variabilities of PDX/CDX models across patients MU150 and MU197, we compared aggregated snRNA-seq of MU150 (PDX + CDX) with MU197 (PDX + CDX). DEGs were strikingly different (Fig. S8 A); GSEA and pathway enrichment analysis showed upregulation of cell adhesion/migration and EMT genes in MU150 PDX/CDX (Fig. S8 B/C) which may be the reason for aggressive tumor growth compared to MU197 PDX/CDX models as discussed above (Fig. S8 G/H). Further, aggregation of CDX models across patients showed that MU150 CDX is different than MU197 CDX (Fig. S8 D). MU150 CDX showed enrichment of know cancer aggressiveness causing genes/pathways, such as cell adhesion/migration genes and ERBB pathway (Fig. S8 E/F), which may be the reason for the aggressive growth kinetics of MU150 CDX versus MU197 CDX (Fig. S8 H).

### Chemosensitivity testing of CDX models with carboplatin/paclitaxel and MYC blockade to overcome drug resistance

**Fig. 2:**
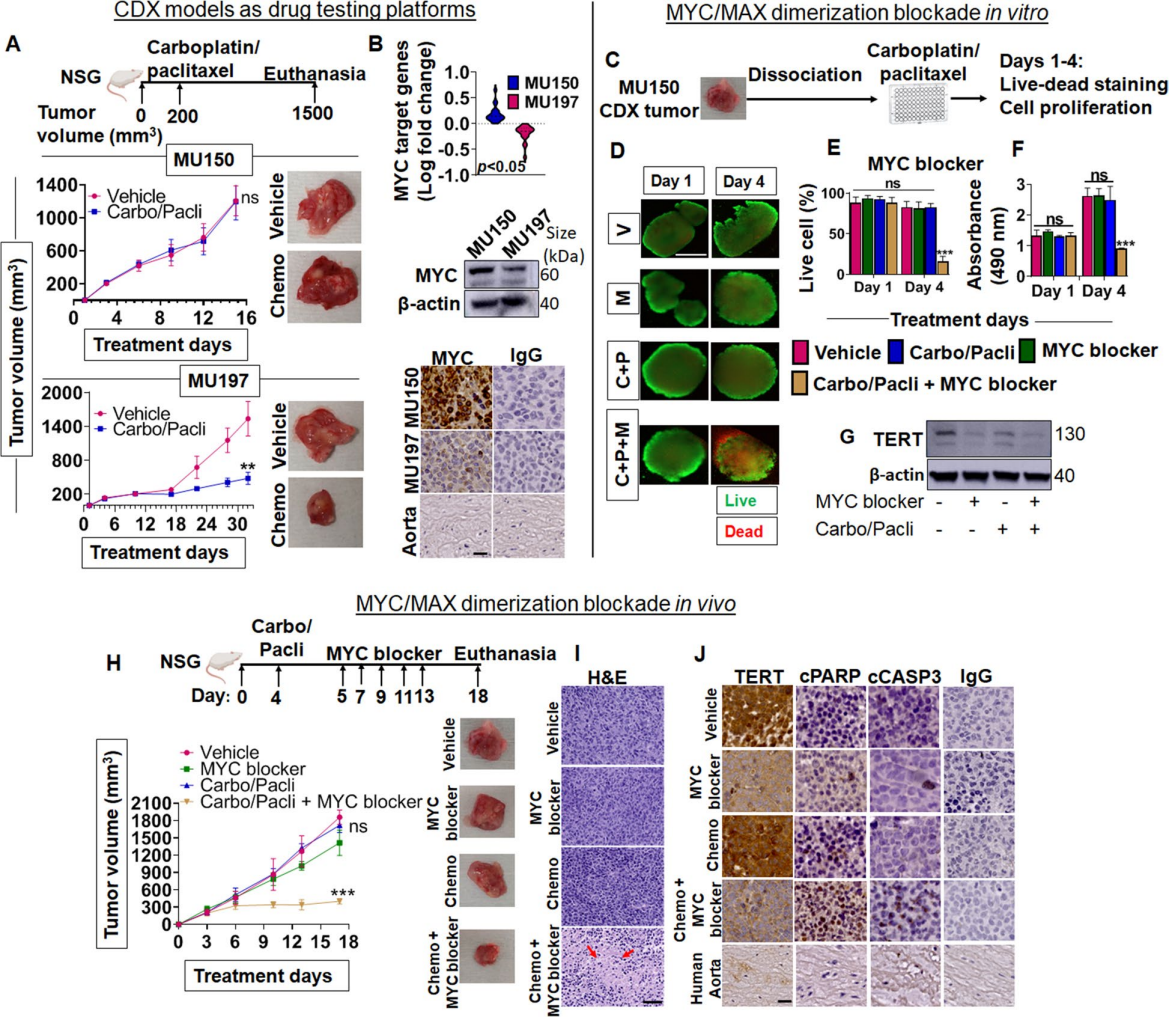
CDX models as drug testing platforms to study responses and overcoming drug resistance by blocking MYC/MAX dimerization: **A** Mice (*n* = 5) bearing MU150 and MU197 CDX tumors were intraperitoneally injected with standard-of-care doublet carboplatin/paclitaxel versus vehicle control. Tumor growth was monitored. Upper panel: Treatment schedules. Middle panel: Tumor growth curves demonstrate that MU150 CDX is resistant towards chemotherapy. Lower panel: Tumor growth curves demonstrate that MU197 CDX is sensitive towards chemotherapy. *n* = 5, error bars represent mean ± standard error of the mean (SEM) (ns-not significant, **p < 0.01; Student’s *t*-test). **B** Differential expression of Hallmark MYC target genes and MYC protein between chemoresistant MU150 CDX versus chemosensitive MU197 CDX. Upper panel: Differential expression between MU150 and MU197 CDX models were obtained by integrating snRNA-seq data sets by MAST algorithm. Violin plots depicting differential log fold change expression of Hallmark MYC targets that were significantly higher in chemoresistant MU150 CDX tumors. Middle panel: Western blots for MYC protein (and β-actin control) in CDX tumor lysates (biological triplicates) show higher MYC expression in chemoresistant MU150. Lower panel: MYC immunostaining shows higher expression in chemoresistant MU150 versus chemosensitive MU197 CDX tumor tissues (human aorta served as negative control tissue, IgG as isotype control). Scale bar, 20 μm. **C** Experimental design of MYC/MAX dimerization blockade in chemotherapy resistant MU150 CDX tumor-derived cells in vitro. **D** CDX tumor-derived cells were cultured and treated with carboplatin/paclitaxel with or without MYC blocker 10058-F4. Live/dead cell staining demonstrates cell death on day 4 in the Carbo/Pacli/MYC blocker (C + P + M) group versus Carbo/Pacli (C + P), MYC blocker alone (M) and vehicle (V) groups, indicating that MYC blockade reverses drug resistance. (4X magnification, scale bar 200 μm). Images represent biological triplicates. **E** Quantification of live/dead cell percentage by hybrid cell count method, and **F** Cell proliferation assay of MU150 showing significant reduction in proliferation in the Carbo/Pacli/MYC blocker group (***p < 0.001, ns-not significant; Multiple *t*-test and significance was determined by Holm-Sidak method; error bars represent mean ± SEM; biological triplicates). **G** Western blots showing direct MYC/MAX dimerization target TERT protein expression inhibition in MYC blocker-treated groups (loading control: β-actin) (biological triplicates). **H** Blocking MYC/MAX dimerization overcomes chemotherapy resistance in MU150 CDX model in vivo. Upper panel: Treatment outline. Mice (*n* = 4) bearing MU150 CDX tumors were treated with doublet carboplatin/paclitaxel (Carbo/Pacli) with or without MYC blocker and MYC blocker alone (vs. vehicle control). Lower panel: Tumor growth graphs and representative tumor images demonstrate that MYC blockade overcomes drug resistance (error bars: ±SEM; ns-not significant, *** *p* < 0.001; two-way ANOVA). **I** H&E staining of representative tumor images for all the groups, with highest degree of necrosis (arrows) in carboplatin/paclitaxel/MYC blocker-treated groups (scale bar, 50 μm). **J** Immunohistochemistry of TERT demonstrate lower expression with MYC/MAX dimerization inhibition, and higher expression of apoptotic markers cPARP and cCASP3 in the group treated with Carbo/Pacli/MYC blocker (human aorta: negative control tissue; IgG: isotype control; Scale bar, 20 μm) Images are representatives from biological triplicates per model

Post-surgery, patients at higher risk receive chemotherapy to eradicate micrometastatic, minimal residual disease. While PDX models represent the primary tumor, CDX models are derived from CTCs and represent micrometastatic disease targeted by adjuvant chemotherapy. The few CDX models that were established so far have been shown to be valuable tools for drug response testing [8, 9, 21]. Hence, the clinical utility of CDX models as drug testing platforms was determined by administering standard-of-care doublet paclitaxel/carboplatin chemotherapy intraperitoneally to CDX tumor-bearing mice. MU150 CDX tumor growth was not altered by carboplatin/paclitaxel in comparison to vehicle-treated controls, consistent with chemoresistance, whereas MU197 CDX tumors were chemosensitive as demonstrated by significant reduction in tumor growth (*p* < 0.01; Student’s *t*-test) (Fig. 2A). As observed in human patients, we noted differential chemotherapy responses further supporting the potential for these models in translational studies.

MYC is a known contributor to drug resistance in cancer [6]. snRNA-seq DEG analysis showed MYC target genes were enriched in chemoresistant MU150 CDX tumors (Fig. 2B; upper panel). Western blot and immunohistochemistry also confirmed overexpression of MYC protein (Fig. 2B; middle and lower panels). In in vitro studies, carboplatin/paclitaxel-resistant MU150 CDX tumor-derived spheroids became chemosensitive when a MYC/MAX dimerization blocker (10058-F4) was added (Fig. 2C/D). MYC blockade led to a significantly higher dead cell percentage and inhibited cell proliferation (*p* < 0.001, multiple *t*-test) (Fig. 2E/F). Blockade of MYC/MAX dimerization with 10058-F4 was confirmed by downregulated protein expression of telomerase reverse transcriptase (TERT), a direct downstream target of MYC/MAX (Fig. 2G) [22]. Based on these in vitro results, MU150 CDX tumor-bearing mice were treated in vivo with carboplatin/paclitaxel and the MYC blocker. MYC blockade overcame chemoresistance in MU150 CDX tumors, whereas MYC blockade alone had no effect on tumor growth (Fig. 2H). A high degree of necrosis (Fig. 2I), overexpression of apoptosis markers cleaved Poly (ADP-ribose) polymerase (cPARP) and cleaved Caspase-3 (cCASP3) in the carboplatin/paclitaxel plus MYC blocker group, and reduced expression of TERT was observed in tumors treated with MYC blocker (Fig. 2J).

Liquid biopsy CDX mouse models generated from non-metastatic NSCLC ptPDX offer valuable drug sensitivity testing platforms, possibly in a time window before patients develop incurable recurrence. They can also be used to study strategies of chemoresistance reversal, such as by MYC inhibition. CDX models also allow identification of distinct single cell heterogeneity that matches non-xenografted NSCLC metastases.

## Conclusions

Liquid biopsy CDX mouse models provide a platform to study progression from non-metastatic to metastatic NSCLC disease. The strategy of using ptPDX models allowed CDX to be established, overcoming the difficulty of developing these models directly from NSCLC patients with limited disease. CTC isolated from ptPDX were phenotypically the same as those isolated from patients, and CDX established from these CTCs expressed diagnostic pathology markers similar to the patient primary tumor. Thus, as we demonstrate above, these CDX models provide an opportunity to study drug responses and targets for radiographically undetectable micrometastases after curative resection - a critical clinical gap [23]. Additionally, the finding of a second AT2-like cell population in both CDX and patient metastases represents an exciting avenue for future research on CTCs and metastasis development.

## Supplementary Information

**Additional file 1: Supplementary Figure 1.** ptPDX-derived CTCs are similar in morphology and traditionally defined CTC marker expression to patient CTCs. CTCs from matched patient and ptPDX blood were enriched on a microfilter and stained with CK 8/18/19-FITC, EpCAM-PE, CD45-Cy5 and DAPI for nuclei identification. Arrowhead showing a white blood cell (WBC) captured with CTC. Scale bar, 10 μm. **Supplementary Figure 2.** Histopathology and immunohistochemistry staining of patient-matched primary tumor, ptPDX and CDX tumor tissues. Immunohistochemical staining of patient-matched primary, ptPDX and CDX tumor tissues. **A:** Representative images of hematoxylin and eosin (H&E) stained and immunostained (CK7 and Napsin A) patient-matched tumor tissues from MU150. **B:** Representative images of H&E stained and immunostained (CK5/6 and p40) patient-matched tumor tissues from MU197. For both sets of staining, human aorta served as negative control tissue and IgG served as isotype control. Scale bar, 20 μm. **Supplementary Figure 3.** Bright field images of extracted nuclei suspension from automated cell counter after staining with trypan blue. Snap-frozen matched ptPDX and CDX tumor tissues were minced on ice and homogenized. Lysate was transferred through 70 μm cell strainer, homogenized again a few strokes, and passed through 40 μm cell strainer. Nuclei were counted after staining with Trypan blue using Countess II FL Automated Cell Counter to check the quality. **Supplementary Figure 4.** Pre-processing and filtering of snRNA-seq data matrix. Cells were filtered based on RNA transcript count, percentage of mitochondrial (mt)/ribosomal (rb) genes, percentage of mouse reads mapped in alignment to combined human-mouse reference genome. Red dotted line indicates the set cut-off applied to remove outliers. **Supplementary Figure 5.** Heatmaps using differentially expressed genes (DEGs) of all clusters of ptPDX and CDX tumor tissues for cell type annotation. DEGs (Supplementary file 1) having *p adj* of less than 0.05 of each cluster expressing cell type canonical markers (Supplementary file 2). DEGs with *p adj* more than 0.05 were zeroed. **Supplementary Figure 6.** Heatmaps using differentially expressed genes (DEGs) of all clusters of patient primary and metastatic tumor tissues for cell type annotation. DEGs having *p adj* of less than 0.05 of each cluster expressing cell type canonical markers (Supplementary file 2). DEGs with *p adj* more than 0.05 were zeroed. **Supplementary Figure 7.** Integration of snRNA-seq to determine similarities and variabilities within the models. A: Volcano plot showing the differentially expressed genes (DEGs) between MU150 PDX versus MU150 CDX. Genes were grouped into upregulated (Up), downregulated (down), normal and not significant based on the average log fold change and adjusted *p* value (*p adj*). B: Gene set enrichment analysis and pathway enrichment analysis performed using top 100 (up/down regulated) DEGs between MU150 PDX versus MU150 CDX. C: Tumor growth kinetics of MU150 PDX and MU150 CDX. D: Volcano plot showing the DEGs between MU197 PDX versus MU197 CDX. E: Gene set enrichment analysis and pathway enrichment analysis performed using top 100 (up/down regulated) DEGs between MU197 PDX versus MU197. F: Tumor growth kinetics of MU197 PDX and MU197 CDX. DEGs after integration are provided in (Supplementary file 3). **Supplementary Figure 8.** Integration of snRNA-seq to determine similarities and differences across the models. A: Volcano plot showing the differentially expressed genes (DEGs) between MU150 (PDX + CDX) versus MU197 (PDX + CDX). Genes were grouped into upregulated (Up), downregulated (down), normal and not significant based on the average log fold change and adjusted p value (*p adj*). B: Heatmap showing top 100 DEGs. C: Gene set enrichment analysis and pathway enrichment analysis performed using top 100 (up/down regulated) DEGs between MU150 (PDX + CDX) versus MU197 (PDX + CDX). D: Volcano plot showing the differentially expressed genes (DEGs) between MU150 CDX versus MU197 CDX. E: Heatmap showing top 100 DEGs. F: Gene set enrichment analysis and pathway enrichment analysis performed using top 100 (up/down regulated) DEGs between MU150 CDX versus MU197 CDX. G: Tumor growth kinetics of MU150/197 PDXs. H: Tumor growth kinetics of MU150/197 CDXs. DEGs after integration are provided in (Supplementary file 3). **Supplementary Figure 9.** Dose response of drugs against MU150 CDX tumor-derived cells. 0.01 × 10^6^ MU150 CDX tumor-derived cells were seeded in 96 well cell culture dish and treated with increasing concentration of MYC/MAX dimerization blocker (10058-F4) and carboplatin/paclitaxel doublet. Cell proliferation was monitored from day 1 to day 4 by performing cell proliferation assay on each day. A: Effect of increasing concentrations of MYC blocker on cell proliferation (absorbance at 490 nm) measured using the CellTiter 96® Aqueous One solution. B: Effect of increasing concentrations of doublet carboplatin/paclitaxel treatments on cell proliferation.

**Additional file 2. **Differentially expressed genes and cell type canonical markers.

**Additional file 3: Supplementary Table 1**. Clinicopathological information and survival status of the NSCLC patients enrolled for ptPDX (*N* = 10) and CDX (*N* = 2) model development. **Supplementary Table 2**. Total counts of ptPDX individual CTCs and CTC clusters that were detected at the time of injection to naïve NSG mice to develop CDX models.

**Additional file 4. **Supplementary methods.

## Data Availability

All data generated or analyzed during this study, if not included in this article and its supplementary information files, are available from the corresponding author on reasonable request.

## Abbreviations

ADP: Adenosine diphosphate
AJCC: American Joint Committee on Cancer
ANOVA: Analysis of Variance
AT1/2: Alveolar epithelial type I/II cell
cCASP3: Cleaved caspase 3
CD: Cluster of differentiation
CDX: Circulating tumor cell-derived xenograft
CK: Cytokeratin
CTC: Circulating tumor cell
Cy5: Cytochrome 5
DAPI: 4′,6-diamidino-2-phenylindole
DEGs: Differentially expressed genes
DMEM: Dulbecco’s Modified Eagle Medium
EpCAM: Epithelial cell adhesion molecule
FDA: United States Food and Drug Administration
FITC: Fluorescein isothiocyanate
GSEA: Gene set enrichment analysis
H&E: Hematoxylin and eosin
IgG: Immunoglobulin G
IHC: Immunohistochemistry
IRB: Institutional Review Board
FDR: False Discovery Rate
MAST: Model-based Analysis of Single-cell Transcriptomics
MAX: MYC-associated protein X
MYC: Myelocytomatosis oncogene
NCT: National Clinical Trial Identifier
NSCLC: Non-small cell lung cancer
NSG: Non-obese, diabetic (NOD), severe combined immunodeficient (SCID) gamma
PARP: Poly (ADP-ribose) polymerase
PBS: Phosphate buffered saline
cPARP: Cleaved PARP
ptPDX: Primary tumor patient-derived xenograft
PE: Phycoerythrin
PET/CT: Positron emission tomography/computed tomography
Rho: Ras homologous
RNA: Ribonucleic Acid
s.c.: Subcutaneous
scRNA-seq: Single-cell ribonucleic acid sequencing
SEM: Standard error of the mean
snRNA-seq: Single-nucleus ribonucleic acid sequencing
SFTPB: Surfactant protein B
TERT: Telomerase reverse transcriptase
UMAP: Uniform Manifold Approximation and Projection
