# Supplementary material for: Tumorigenic circulating tumor cells from xenograft mouse models of non-metastatic NSCLC patients reveal distinct single cell heterogeneity and drug responses
Source: Mol Cancer. 2022 Mar 12;21:73. doi: 10.1186/s12943-022-01553-5 (PMC8917773; doi:10.1186/s12943-022-01553-5)
Supplement: Supplementary file 1 — Additional file 1: Supplementary Figure 1. ptPDX-derived CTCs are similar in morphology and traditionally defined CTC marker expression to patient CTCs. CTCs from matched patient and ptPDX blood were enriched on a microfilter and stained with CK 8/18/19-FITC, EpCAM-PE, CD45-Cy5 and DAPI for nuclei identification. Arrowhead showing a white blood cell (WBC) captured with CTC. Scale bar, 10 μm. Supplementary Figure 2. Histopathology and immunohistochemistry staining of patient-matched primary tumor, ptPDX and CDX tumor tissues. Immunohistochemical staining of patient-matched primary, ptPDX and CDX tumor tissues. A: Representative images of hematoxylin and eosin (H&E) stained and immunostained (CK7 and Napsin A) patient-matched tumor tissues from MU150. B: Representative images of H&E stained and immunostained (CK5/6 and p40) patient-matched tumor tissues from MU197. For both sets of staining, human aorta served as negative control tissue and IgG served as isotype control. Scale bar, 20 μm. Supplementary Figure 3. Bright field images of extracted nuclei suspension from automated cell counter after staining with trypan blue. Snap-frozen matched ptPDX and CDX tumor tissues were minced on ice and homogenized. Lysate was transferred through 70 μm cell strainer, homogenized again a few strokes, and passed through 40 μm cell strainer. Nuclei were counted after staining with Trypan blue using Countess II FL Automated Cell Counter to check the quality. Supplementary Figure 4. Pre-processing and filtering of snRNA-seq data matrix. Cells were filtered based on RNA transcript count, percentage of mitochondrial (mt)/ribosomal (rb) genes, percentage of mouse reads mapped in alignment to combined human-mouse reference genome. Red dotted line indicates the set cut-off applied to remove outliers. Supplementary Figure 5. Heatmaps using differentially expressed genes (DEGs) of all clusters of ptPDX and CDX tumor tissues for cell type annotation. DEGs (Supplementary file 1) having p adj o [file 12943_2022_1553_MOESM1_ESM.zip › 12943_2022_1553_MOESM1_ESM/Supplementary figures and legends.docx]

**SUPPLEMENTARY FIGURES AND LEGENDS**

**
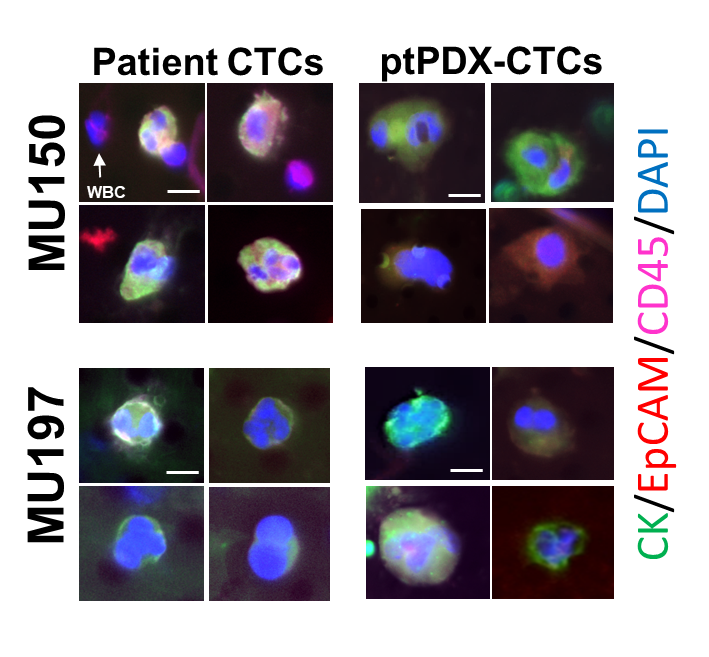
**

**Supplementary Figure 1. ptPDX-derived CTCs are similar in morphology and traditionally defined CTC marker expression to patient CTCs.** CTCs from matched patient and ptPDX blood were enriched on a microfilter and stained with CK 8/18/19-FITC, EpCAM-PE, CD45-Cy5 and DAPI for nuclei identification. Arrowhead showing a white blood cell (WBC) captured with CTC. Scale bar, 10 μm.

**
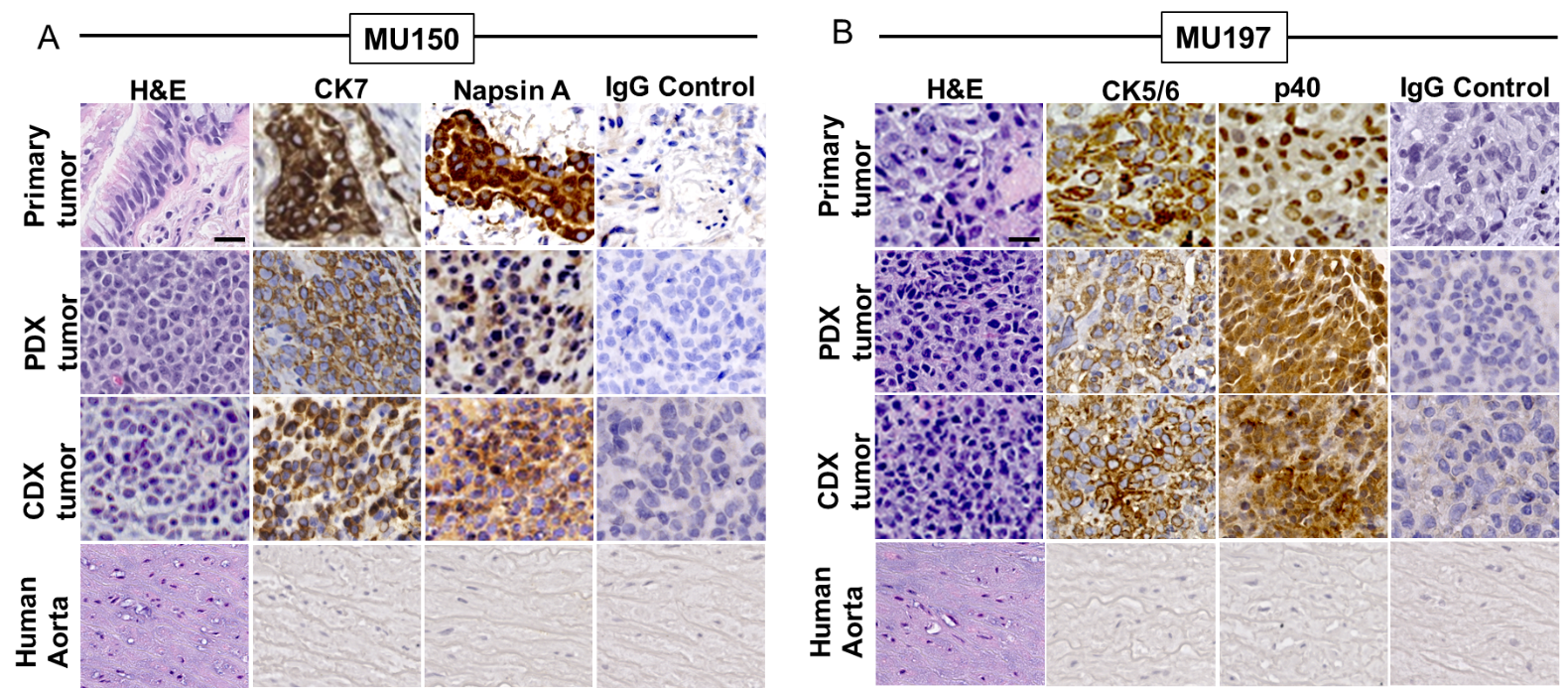
**

**Supplementary Figure 2. Histopathology and immunohistochemistry staining of patient-matched primary tumor, ptPDX and CDX tumor tissues**. Immunohistochemical staining of patient-matched primary, ptPDX and CDX tumor tissues. **A:** Representative images of hematoxylin and eosin (H&E) stained and immunostained (CK7 and Napsin A) patient-matched tumor tissues from MU150. **B:** Representative images of H&E stained and immunostained (CK5/6 and p40) patient-matched tumor tissues from MU197. For both sets of staining, human aorta served as negative control tissue and IgG served as isotype control. Scale bar, 20 μm.


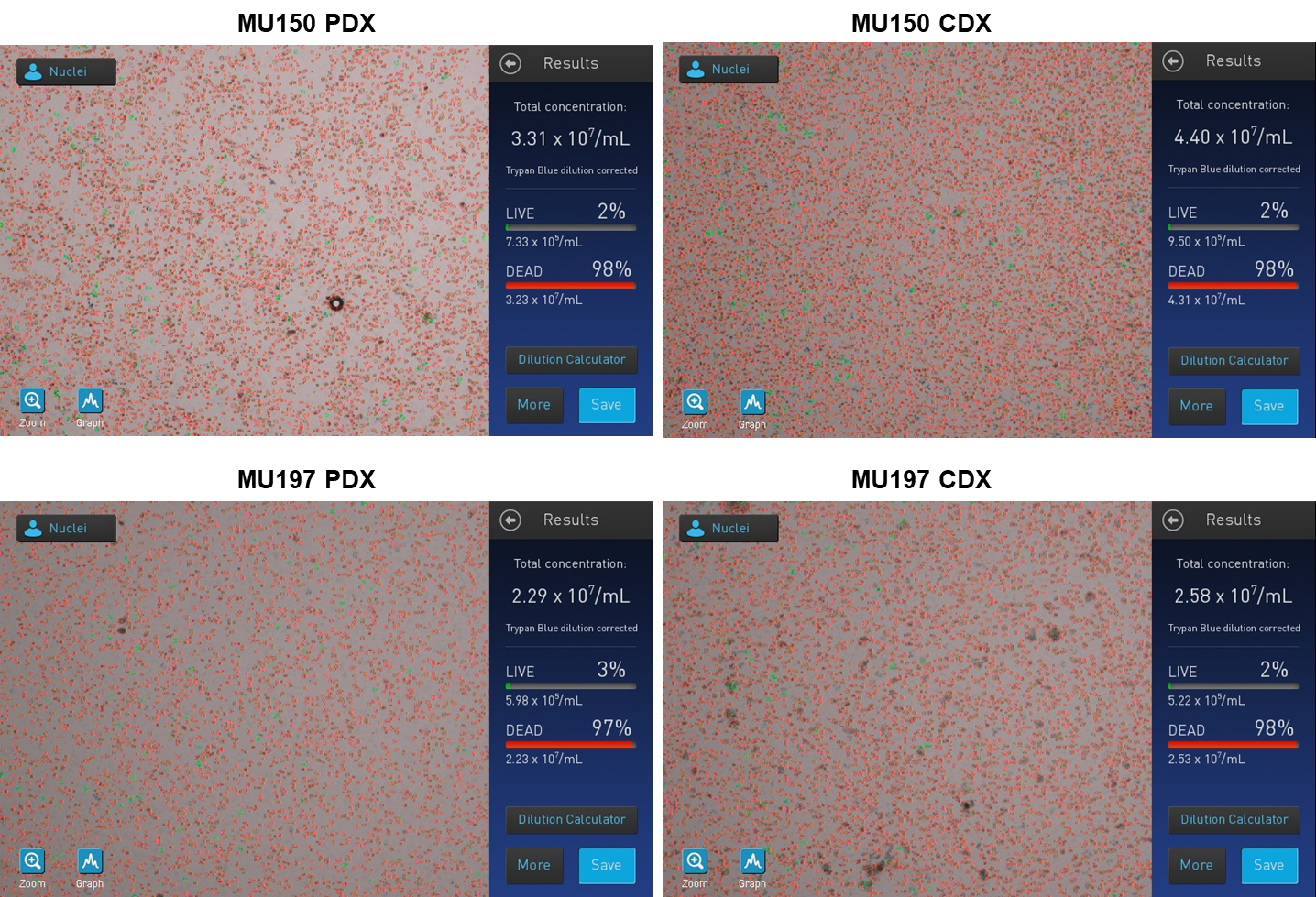


**Supplementary Figure 3**. **Bright field images of extracted nuclei suspension from automated cell counter after staining with trypan blue.** Snap-frozen matched ptPDX and CDX tumor tissues were minced on ice and homogenized. Lysate was transferred through 70 µm cell strainer, homogenized again a few strokes, and passed through 40 µm cell strainer. Nuclei were counted after staining with Trypan blue using Countess II FL Automated Cell Counter to check the quality.


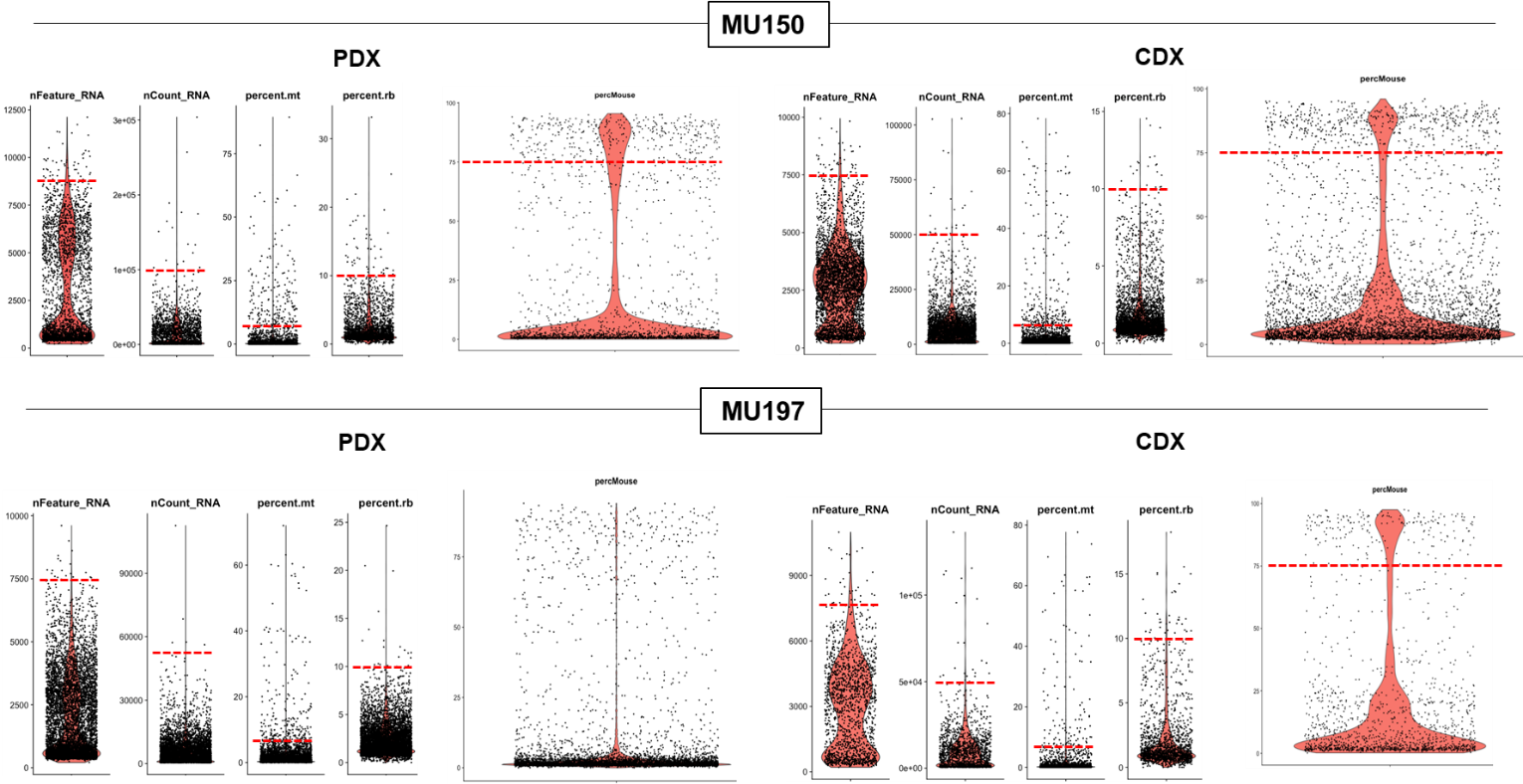


**Supplementary Figure 4.** **Pre-processing and filtering of snRNA-seq data matrix**. Cells were filtered based on RNA transcript count, percentage of mitochondrial (mt)/ribosomal (rb) genes, percentage of mouse reads mapped in alignment to combined human-mouse reference genome. Red dotted line indicates the set cut-off applied to remove outliers.


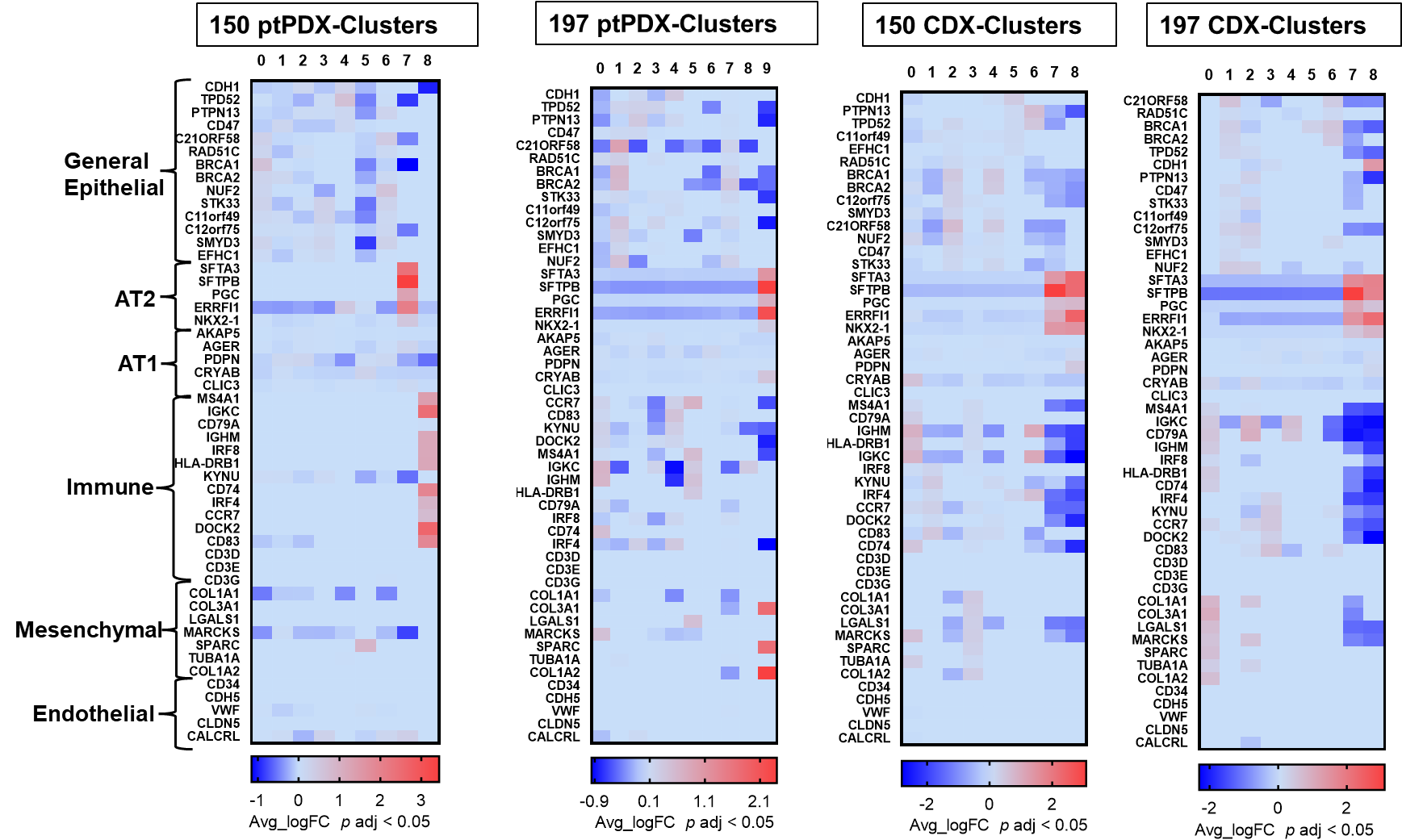


**Supplementary Figure 5. Heatmaps using differentially expressed genes (DEGs) of all clusters of ptPDX and CDX tumor tissues for cell type annotation**. DEGs (Supplementary file 1) having *p adj* of less than 0.05 of each cluster expressing cell type canonical markers (Supplementary file 2). DEGs with *p adj* more than 0.05 were zeroed.


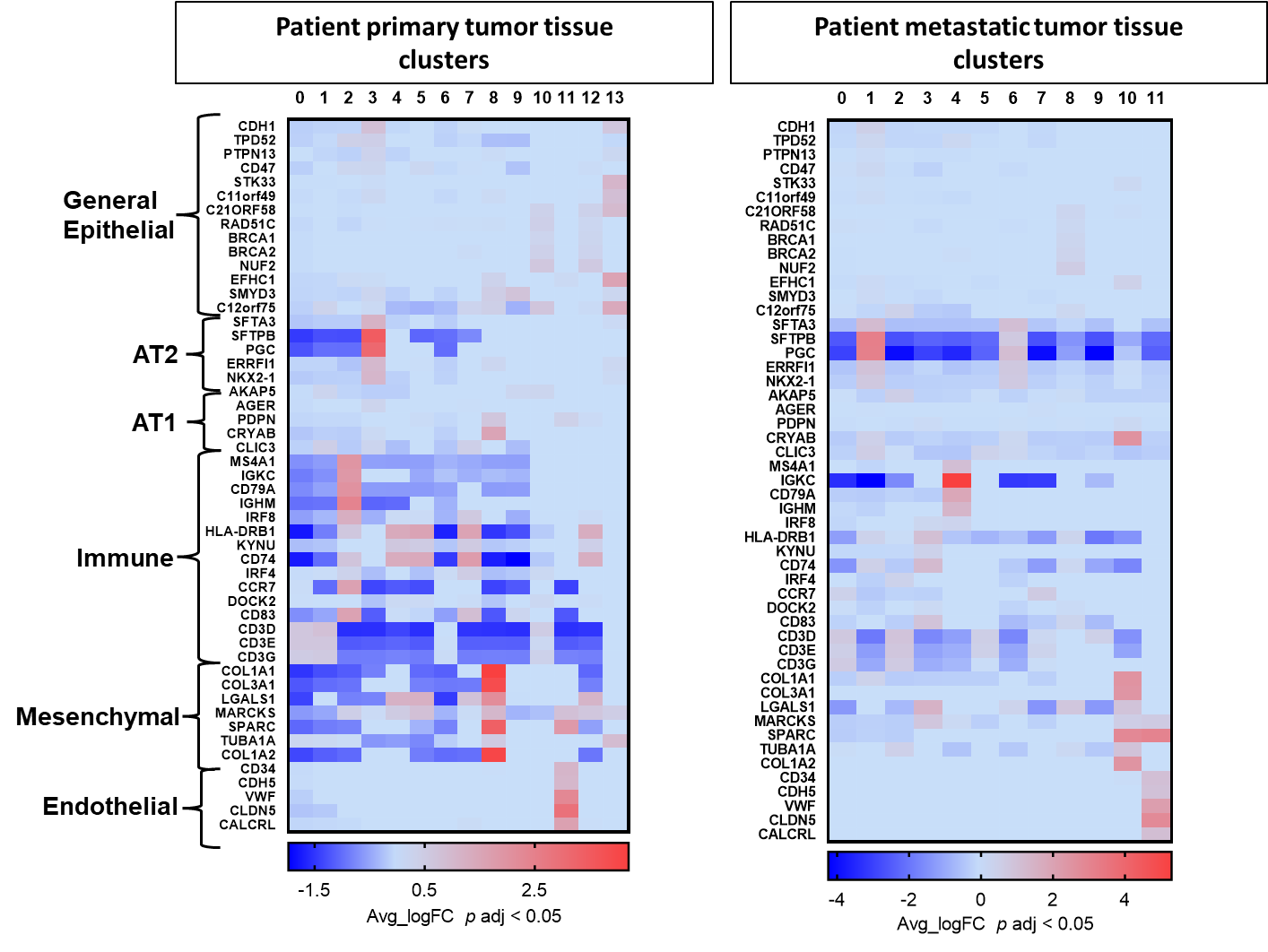


**Supplementary Figure 6. Heatmaps using differentially expressed genes (DEGs) of all clusters of patient primary and metastatic tumor tissues for cell type annotation**. DEGs having *p adj* of less than 0.05 of each cluster expressing cell type canonical markers (Supplementary file 2). DEGs with *p adj* more than 0.05 were zeroed.


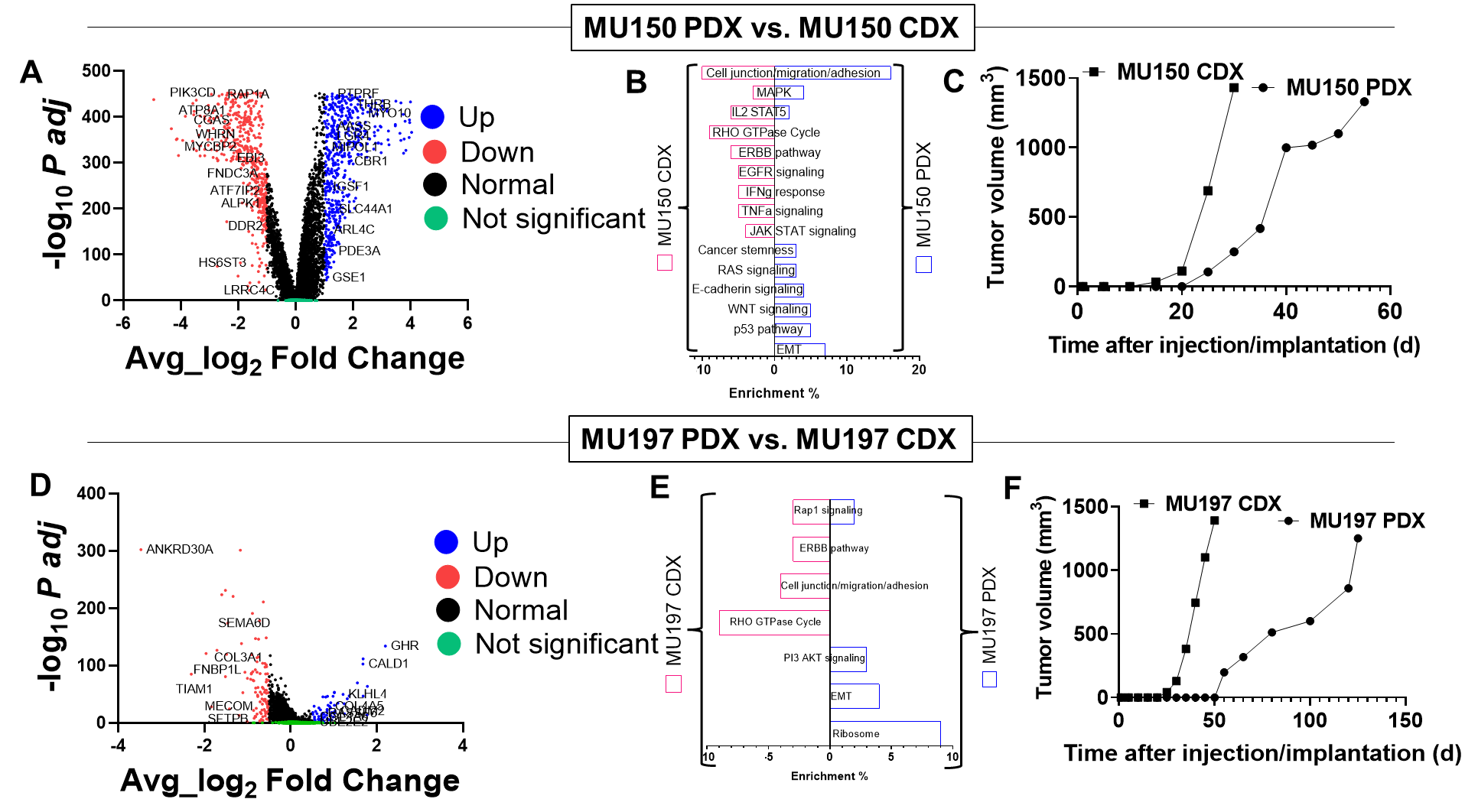


**Supplementary Figure 7. Integration of snRNA-seq to determine similarities and variabilities within the models**. A: Volcano plot showing the differentially expressed genes (DEGs) between MU150 PDX versus MU150 CDX. Genes were grouped into upregulated (Up), downregulated (down), normal and not significant based on the average log fold change and adjusted *p* value (*p adj*). B: Gene set enrichment analysis and pathway enrichment analysis performed using top 100 (up/down regulated) DEGs between MU150 PDX versus MU150 CDX. C: Tumor growth kinetics of MU150 PDX and MU150 CDX. D: Volcano plot showing the DEGs between MU197 PDX versus MU197 CDX. E: Gene set enrichment analysis and pathway enrichment analysis performed using top 100 (up/down regulated) DEGs between MU197 PDX versus MU197. F: Tumor growth kinetics of MU197 PDX and MU197 CDX. DEGs after integration are provided in (Supplementary file 3).


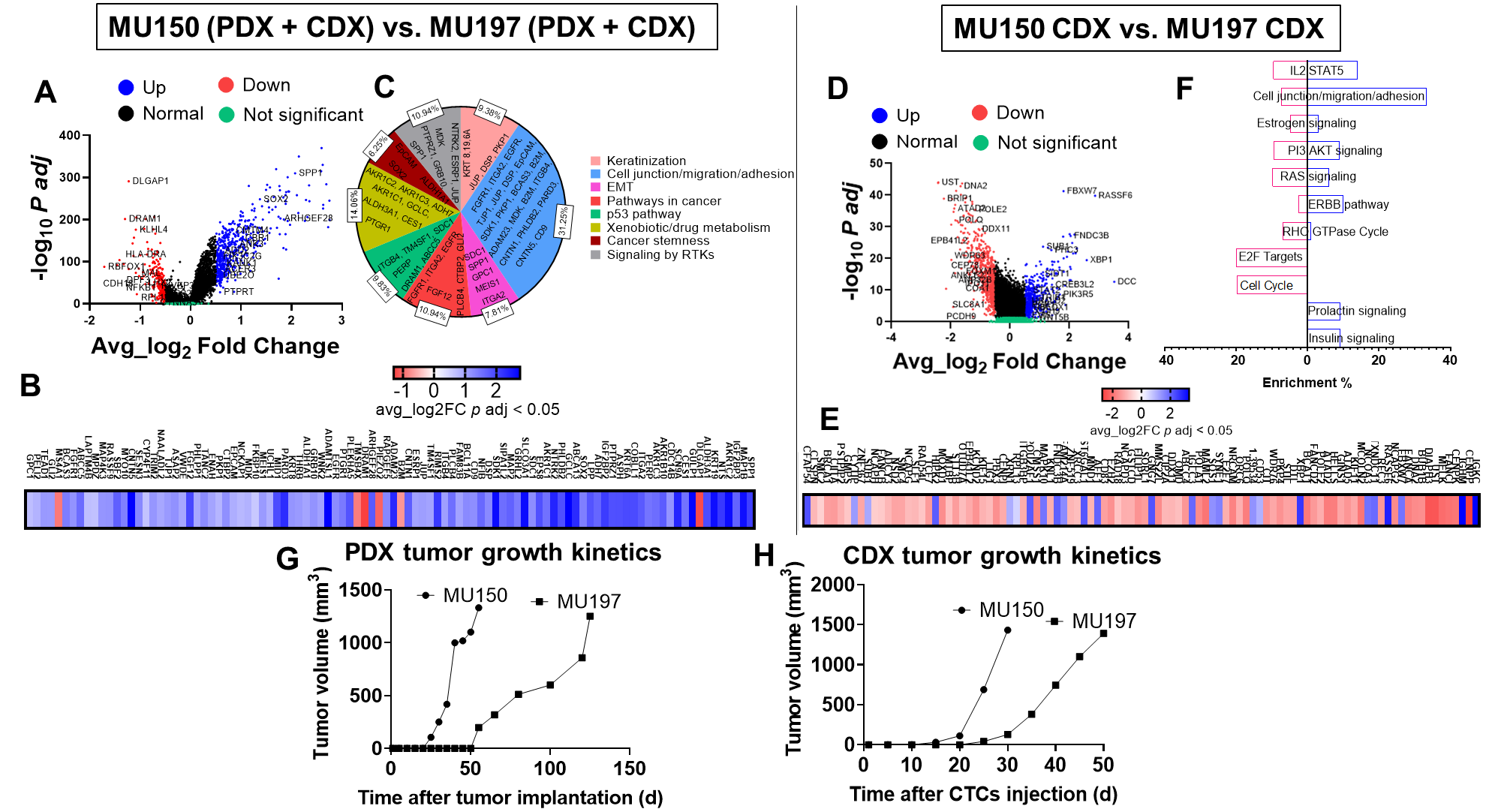


**Supplementary Figure 8. Integration of snRNA-seq to determine similarities and differences across the models.** A: Volcano plot showing the differentially expressed genes (DEGs) between MU150 (PDX+CDX) versus MU197 (PDX+CDX). Genes were grouped into upregulated (Up), downregulated (down), normal and not significant based on the average log fold change and adjusted p value (*p adj*). B: Heatmap showing top 100 DEGs. C: Gene set enrichment analysis and pathway enrichment analysis performed using top 100 (up/down regulated) DEGs between MU150 (PDX+CDX) versus MU197 (PDX+CDX). D: Volcano plot showing the differentially expressed genes (DEGs) between MU150 CDX versus MU197 CDX. E: Heatmap showing top 100 DEGs. F: Gene set enrichment analysis and pathway enrichment analysis performed using top 100 (up/down regulated) DEGs between MU150 CDX versus MU197 CDX. G: Tumor growth kinetics of MU150/197 PDXs. H: Tumor growth kinetics of MU150/197 CDXs. DEGs after integration are provided in (Supplementary file 3).

**
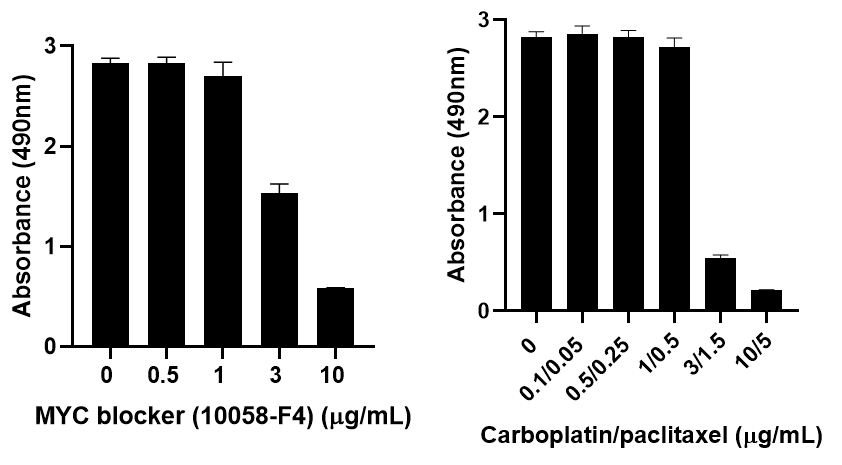
Supplementary Figure 9. Dose response of drugs against MU150 CDX tumor-derived cells**. 0.01 × 10^6^ MU150 CDX tumor-derived cells were seeded in 96 well cell culture dish and treated with increasing concentration of MYC/MAX dimerization blocker (10058-F4) and carboplatin/paclitaxel doublet. Cell proliferation was monitored from day 1 to day 4 by performing cell proliferation assay on each day. A: Effect of increasing concentrations of MYC blocker on cell proliferation (absorbance at 490 nm) measured using the CellTiter 96^®^ Aqueous One solution. B: Effect of increasing concentrations of doublet carboplatin/paclitaxel treatments on cell proliferation.
