# Supplementary figures and images for: Tumorigenic circulating tumor cells from xenograft mouse models of non-metastatic NSCLC patients reveal distinct single cell heterogeneity and drug responses
Source: Mol Cancer. 2022 Mar 12;21:73. doi: 10.1186/s12943-022-01553-5 (PMC8917773; doi:10.1186/s12943-022-01553-5)

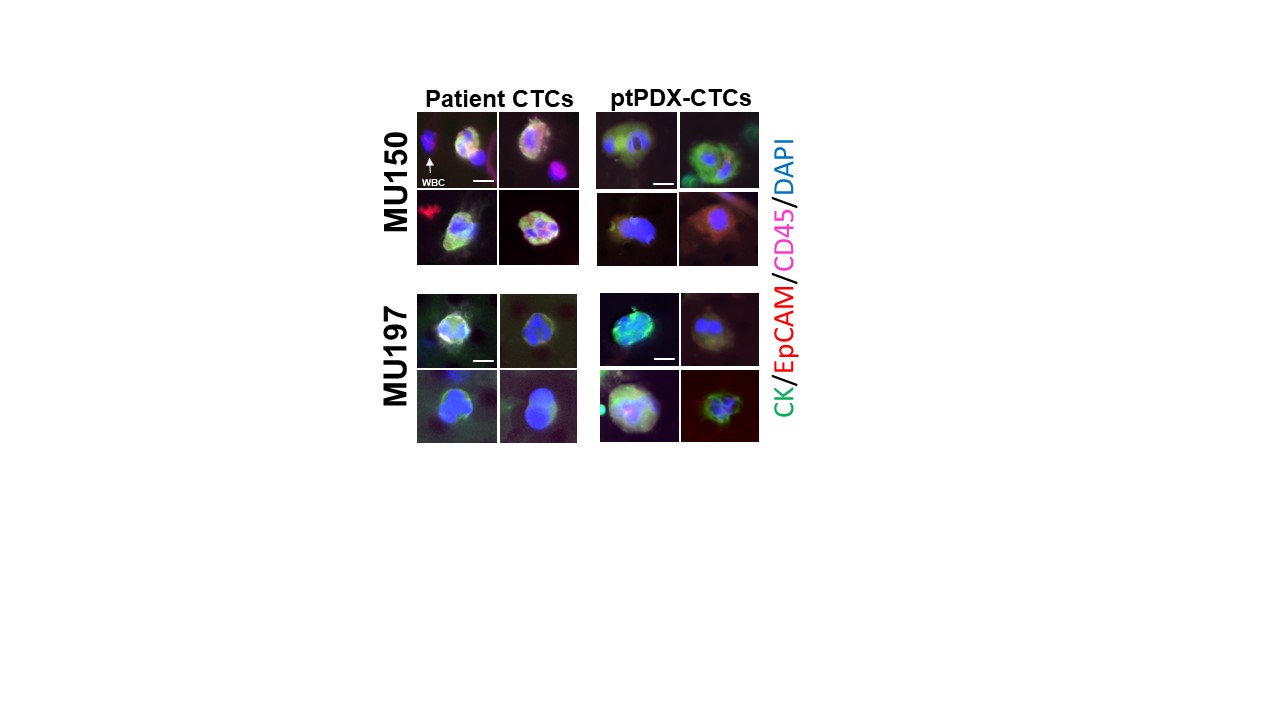

Supplement: Supplementary file 1 — Additional file 1: Supplementary Figure 1. ptPDX-derived CTCs are similar in morphology and traditionally defined CTC marker expression to patient CTCs. CTCs from matched patient and ptPDX blood were enriched on a microfilter and stained with CK 8/18/19-FITC, EpCAM-PE, CD45-Cy5 and DAPI for nuclei identification. Arrowhead showing a white blood cell (WBC) captured with CTC. Scale bar, 10 μm. Supplementary Figure 2. Histopathology and immunohistochemistry staining of patient-matched primary tumor, ptPDX and CDX tumor tissues. Immunohistochemical staining of patient-matched primary, ptPDX and CDX tumor tissues. A: Representative images of hematoxylin and eosin (H&E) stained and immunostained (CK7 and Napsin A) patient-matched tumor tissues from MU150. B: Representative images of H&E stained and immunostained (CK5/6 and p40) patient-matched tumor tissues from MU197. For both sets of staining, human aorta served as negative control tissue and IgG served as isotype control. Scale bar, 20 μm. Supplementary Figure 3. Bright field images of extracted nuclei suspension from automated cell counter after staining with trypan blue. Snap-frozen matched ptPDX and CDX tumor tissues were minced on ice and homogenized. Lysate was transferred through 70 μm cell strainer, homogenized again a few strokes, and passed through 40 μm cell strainer. Nuclei were counted after staining with Trypan blue using Countess II FL Automated Cell Counter to check the quality. Supplementary Figure 4. Pre-processing and filtering of snRNA-seq data matrix. Cells were filtered based on RNA transcript count, percentage of mitochondrial (mt)/ribosomal (rb) genes, percentage of mouse reads mapped in alignment to combined human-mouse reference genome. Red dotted line indicates the set cut-off applied to remove outliers. Supplementary Figure 5. Heatmaps using differentially expressed genes (DEGs) of all clusters of ptPDX and CDX tumor tissues for cell type annotation. DEGs (Supplementary file 1) having p adj o [file 12943_2022_1553_MOESM1_ESM.zip › 12943_2022_1553_MOESM1_ESM/Supplementary Figure 1.jpg]

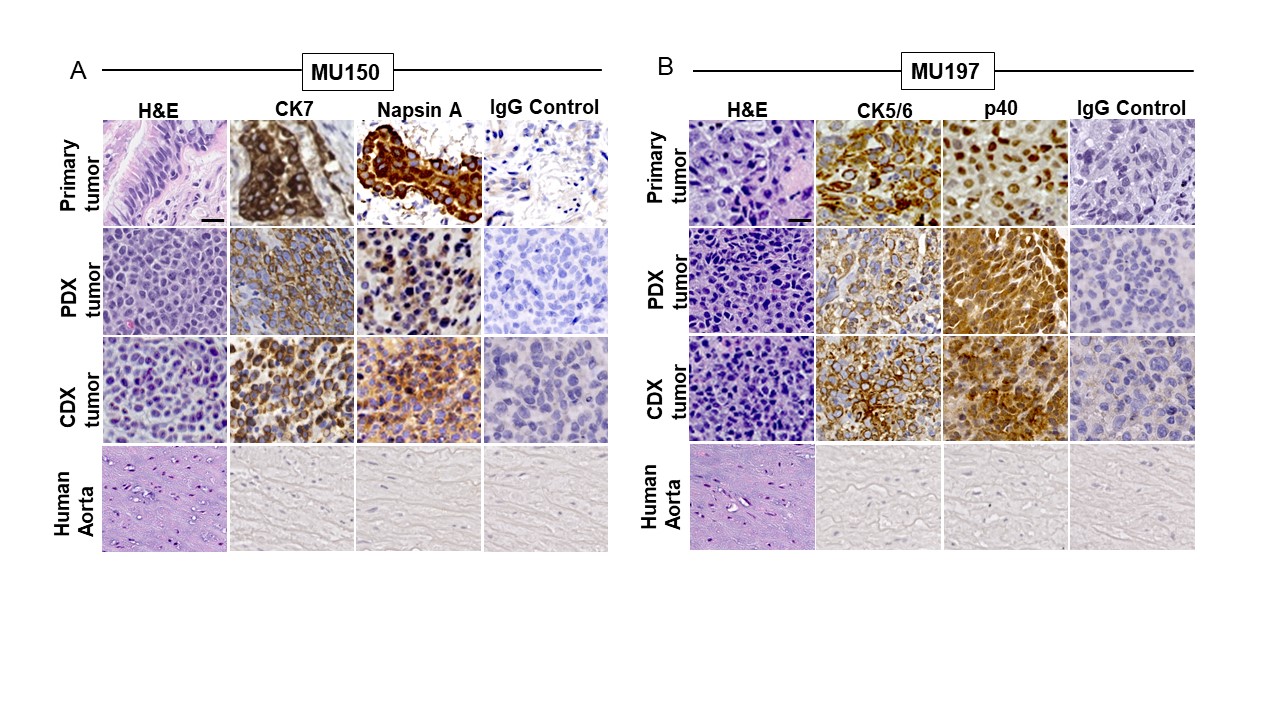

Supplement: Supplementary file 1 — Additional file 1: Supplementary Figure 1. ptPDX-derived CTCs are similar in morphology and traditionally defined CTC marker expression to patient CTCs. CTCs from matched patient and ptPDX blood were enriched on a microfilter and stained with CK 8/18/19-FITC, EpCAM-PE, CD45-Cy5 and DAPI for nuclei identification. Arrowhead showing a white blood cell (WBC) captured with CTC. Scale bar, 10 μm. Supplementary Figure 2. Histopathology and immunohistochemistry staining of patient-matched primary tumor, ptPDX and CDX tumor tissues. Immunohistochemical staining of patient-matched primary, ptPDX and CDX tumor tissues. A: Representative images of hematoxylin and eosin (H&E) stained and immunostained (CK7 and Napsin A) patient-matched tumor tissues from MU150. B: Representative images of H&E stained and immunostained (CK5/6 and p40) patient-matched tumor tissues from MU197. For both sets of staining, human aorta served as negative control tissue and IgG served as isotype control. Scale bar, 20 μm. Supplementary Figure 3. Bright field images of extracted nuclei suspension from automated cell counter after staining with trypan blue. Snap-frozen matched ptPDX and CDX tumor tissues were minced on ice and homogenized. Lysate was transferred through 70 μm cell strainer, homogenized again a few strokes, and passed through 40 μm cell strainer. Nuclei were counted after staining with Trypan blue using Countess II FL Automated Cell Counter to check the quality. Supplementary Figure 4. Pre-processing and filtering of snRNA-seq data matrix. Cells were filtered based on RNA transcript count, percentage of mitochondrial (mt)/ribosomal (rb) genes, percentage of mouse reads mapped in alignment to combined human-mouse reference genome. Red dotted line indicates the set cut-off applied to remove outliers. Supplementary Figure 5. Heatmaps using differentially expressed genes (DEGs) of all clusters of ptPDX and CDX tumor tissues for cell type annotation. DEGs (Supplementary file 1) having p adj o [file 12943_2022_1553_MOESM1_ESM.zip › 12943_2022_1553_MOESM1_ESM/Supplementary Figure 2.jpg]

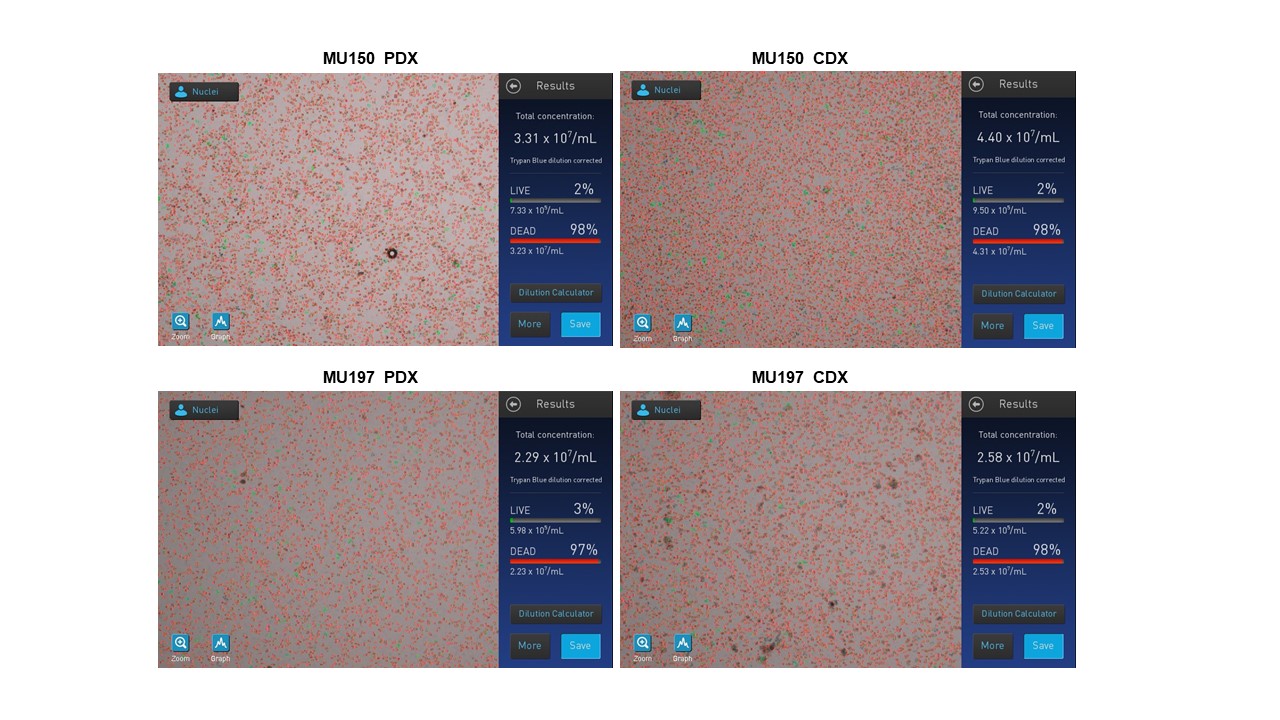

Supplement: Supplementary file 1 — Additional file 1: Supplementary Figure 1. ptPDX-derived CTCs are similar in morphology and traditionally defined CTC marker expression to patient CTCs. CTCs from matched patient and ptPDX blood were enriched on a microfilter and stained with CK 8/18/19-FITC, EpCAM-PE, CD45-Cy5 and DAPI for nuclei identification. Arrowhead showing a white blood cell (WBC) captured with CTC. Scale bar, 10 μm. Supplementary Figure 2. Histopathology and immunohistochemistry staining of patient-matched primary tumor, ptPDX and CDX tumor tissues. Immunohistochemical staining of patient-matched primary, ptPDX and CDX tumor tissues. A: Representative images of hematoxylin and eosin (H&E) stained and immunostained (CK7 and Napsin A) patient-matched tumor tissues from MU150. B: Representative images of H&E stained and immunostained (CK5/6 and p40) patient-matched tumor tissues from MU197. For both sets of staining, human aorta served as negative control tissue and IgG served as isotype control. Scale bar, 20 μm. Supplementary Figure 3. Bright field images of extracted nuclei suspension from automated cell counter after staining with trypan blue. Snap-frozen matched ptPDX and CDX tumor tissues were minced on ice and homogenized. Lysate was transferred through 70 μm cell strainer, homogenized again a few strokes, and passed through 40 μm cell strainer. Nuclei were counted after staining with Trypan blue using Countess II FL Automated Cell Counter to check the quality. Supplementary Figure 4. Pre-processing and filtering of snRNA-seq data matrix. Cells were filtered based on RNA transcript count, percentage of mitochondrial (mt)/ribosomal (rb) genes, percentage of mouse reads mapped in alignment to combined human-mouse reference genome. Red dotted line indicates the set cut-off applied to remove outliers. Supplementary Figure 5. Heatmaps using differentially expressed genes (DEGs) of all clusters of ptPDX and CDX tumor tissues for cell type annotation. DEGs (Supplementary file 1) having p adj o [file 12943_2022_1553_MOESM1_ESM.zip › 12943_2022_1553_MOESM1_ESM/Supplementary Figure 3.jpg]

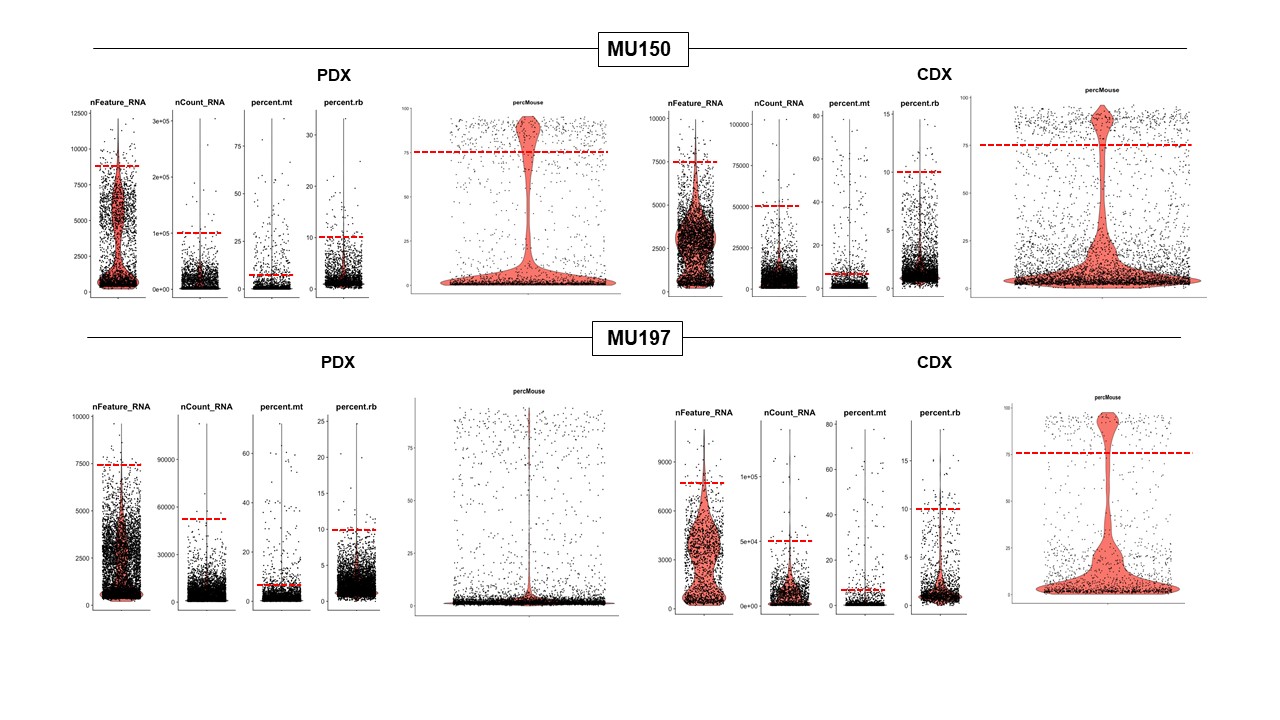

Supplement: Supplementary file 1 — Additional file 1: Supplementary Figure 1. ptPDX-derived CTCs are similar in morphology and traditionally defined CTC marker expression to patient CTCs. CTCs from matched patient and ptPDX blood were enriched on a microfilter and stained with CK 8/18/19-FITC, EpCAM-PE, CD45-Cy5 and DAPI for nuclei identification. Arrowhead showing a white blood cell (WBC) captured with CTC. Scale bar, 10 μm. Supplementary Figure 2. Histopathology and immunohistochemistry staining of patient-matched primary tumor, ptPDX and CDX tumor tissues. Immunohistochemical staining of patient-matched primary, ptPDX and CDX tumor tissues. A: Representative images of hematoxylin and eosin (H&E) stained and immunostained (CK7 and Napsin A) patient-matched tumor tissues from MU150. B: Representative images of H&E stained and immunostained (CK5/6 and p40) patient-matched tumor tissues from MU197. For both sets of staining, human aorta served as negative control tissue and IgG served as isotype control. Scale bar, 20 μm. Supplementary Figure 3. Bright field images of extracted nuclei suspension from automated cell counter after staining with trypan blue. Snap-frozen matched ptPDX and CDX tumor tissues were minced on ice and homogenized. Lysate was transferred through 70 μm cell strainer, homogenized again a few strokes, and passed through 40 μm cell strainer. Nuclei were counted after staining with Trypan blue using Countess II FL Automated Cell Counter to check the quality. Supplementary Figure 4. Pre-processing and filtering of snRNA-seq data matrix. Cells were filtered based on RNA transcript count, percentage of mitochondrial (mt)/ribosomal (rb) genes, percentage of mouse reads mapped in alignment to combined human-mouse reference genome. Red dotted line indicates the set cut-off applied to remove outliers. Supplementary Figure 5. Heatmaps using differentially expressed genes (DEGs) of all clusters of ptPDX and CDX tumor tissues for cell type annotation. DEGs (Supplementary file 1) having p adj o [file 12943_2022_1553_MOESM1_ESM.zip › 12943_2022_1553_MOESM1_ESM/Supplementary Figure 4.jpg]

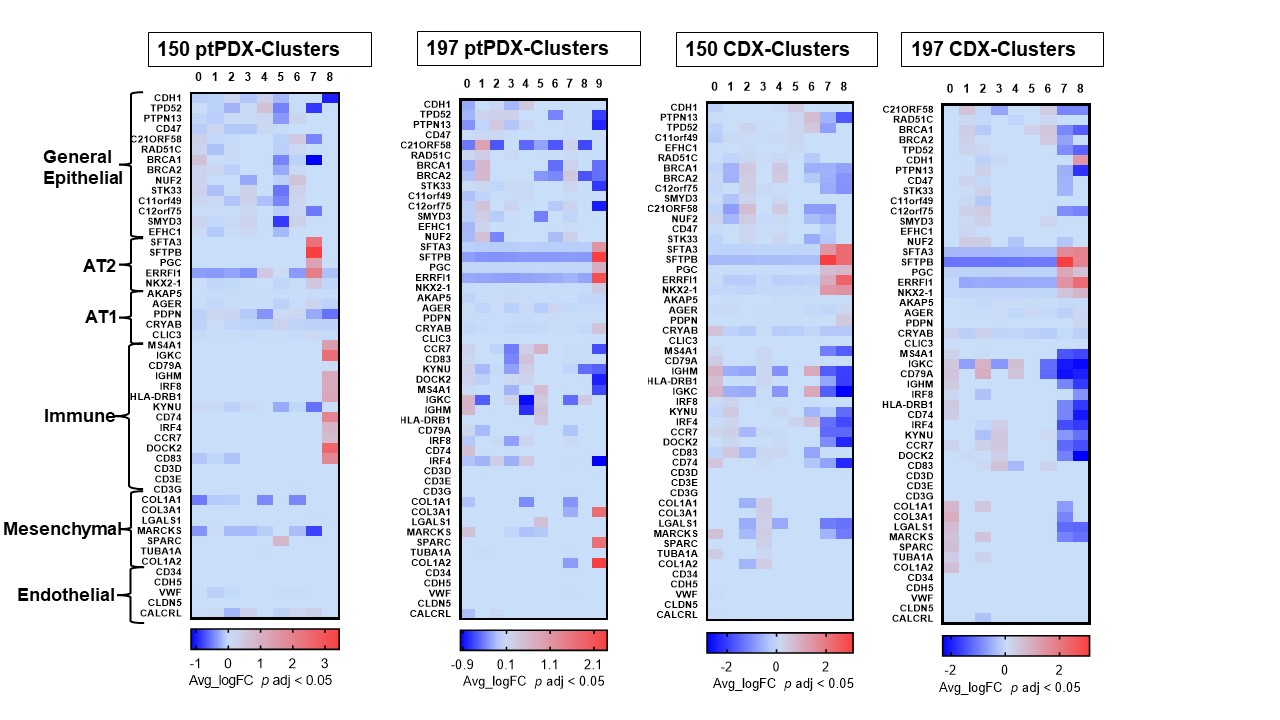

Supplement: Supplementary file 1 — Additional file 1: Supplementary Figure 1. ptPDX-derived CTCs are similar in morphology and traditionally defined CTC marker expression to patient CTCs. CTCs from matched patient and ptPDX blood were enriched on a microfilter and stained with CK 8/18/19-FITC, EpCAM-PE, CD45-Cy5 and DAPI for nuclei identification. Arrowhead showing a white blood cell (WBC) captured with CTC. Scale bar, 10 μm. Supplementary Figure 2. Histopathology and immunohistochemistry staining of patient-matched primary tumor, ptPDX and CDX tumor tissues. Immunohistochemical staining of patient-matched primary, ptPDX and CDX tumor tissues. A: Representative images of hematoxylin and eosin (H&E) stained and immunostained (CK7 and Napsin A) patient-matched tumor tissues from MU150. B: Representative images of H&E stained and immunostained (CK5/6 and p40) patient-matched tumor tissues from MU197. For both sets of staining, human aorta served as negative control tissue and IgG served as isotype control. Scale bar, 20 μm. Supplementary Figure 3. Bright field images of extracted nuclei suspension from automated cell counter after staining with trypan blue. Snap-frozen matched ptPDX and CDX tumor tissues were minced on ice and homogenized. Lysate was transferred through 70 μm cell strainer, homogenized again a few strokes, and passed through 40 μm cell strainer. Nuclei were counted after staining with Trypan blue using Countess II FL Automated Cell Counter to check the quality. Supplementary Figure 4. Pre-processing and filtering of snRNA-seq data matrix. Cells were filtered based on RNA transcript count, percentage of mitochondrial (mt)/ribosomal (rb) genes, percentage of mouse reads mapped in alignment to combined human-mouse reference genome. Red dotted line indicates the set cut-off applied to remove outliers. Supplementary Figure 5. Heatmaps using differentially expressed genes (DEGs) of all clusters of ptPDX and CDX tumor tissues for cell type annotation. DEGs (Supplementary file 1) having p adj o [file 12943_2022_1553_MOESM1_ESM.zip › 12943_2022_1553_MOESM1_ESM/Supplementary Figure 5.jpg]

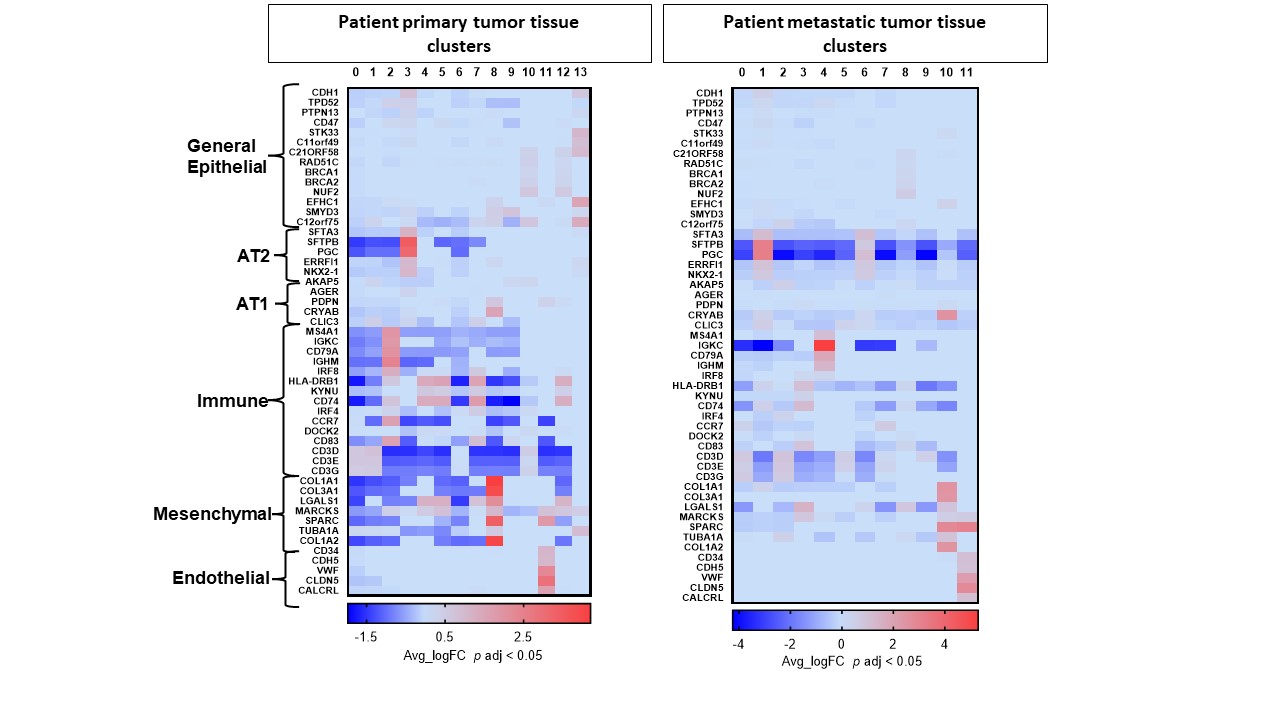

Supplement: Supplementary file 1 — Additional file 1: Supplementary Figure 1. ptPDX-derived CTCs are similar in morphology and traditionally defined CTC marker expression to patient CTCs. CTCs from matched patient and ptPDX blood were enriched on a microfilter and stained with CK 8/18/19-FITC, EpCAM-PE, CD45-Cy5 and DAPI for nuclei identification. Arrowhead showing a white blood cell (WBC) captured with CTC. Scale bar, 10 μm. Supplementary Figure 2. Histopathology and immunohistochemistry staining of patient-matched primary tumor, ptPDX and CDX tumor tissues. Immunohistochemical staining of patient-matched primary, ptPDX and CDX tumor tissues. A: Representative images of hematoxylin and eosin (H&E) stained and immunostained (CK7 and Napsin A) patient-matched tumor tissues from MU150. B: Representative images of H&E stained and immunostained (CK5/6 and p40) patient-matched tumor tissues from MU197. For both sets of staining, human aorta served as negative control tissue and IgG served as isotype control. Scale bar, 20 μm. Supplementary Figure 3. Bright field images of extracted nuclei suspension from automated cell counter after staining with trypan blue. Snap-frozen matched ptPDX and CDX tumor tissues were minced on ice and homogenized. Lysate was transferred through 70 μm cell strainer, homogenized again a few strokes, and passed through 40 μm cell strainer. Nuclei were counted after staining with Trypan blue using Countess II FL Automated Cell Counter to check the quality. Supplementary Figure 4. Pre-processing and filtering of snRNA-seq data matrix. Cells were filtered based on RNA transcript count, percentage of mitochondrial (mt)/ribosomal (rb) genes, percentage of mouse reads mapped in alignment to combined human-mouse reference genome. Red dotted line indicates the set cut-off applied to remove outliers. Supplementary Figure 5. Heatmaps using differentially expressed genes (DEGs) of all clusters of ptPDX and CDX tumor tissues for cell type annotation. DEGs (Supplementary file 1) having p adj o [file 12943_2022_1553_MOESM1_ESM.zip › 12943_2022_1553_MOESM1_ESM/Supplementary Figure 6.jpg]

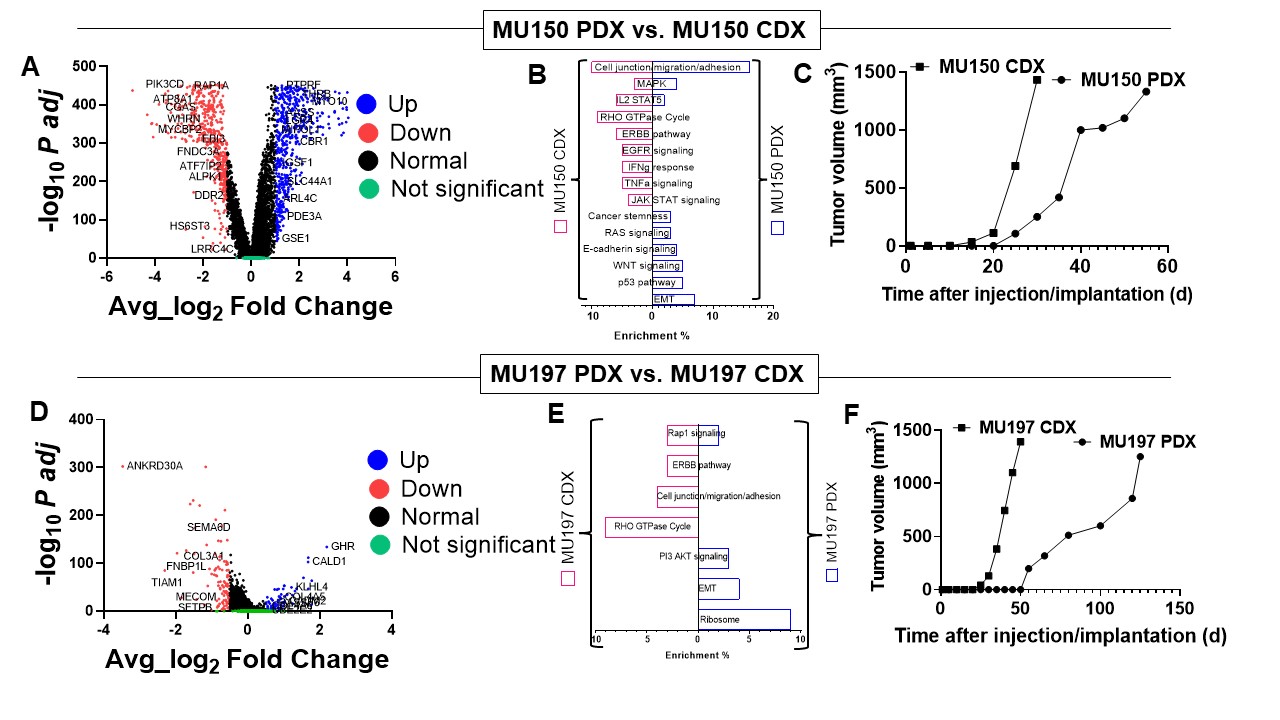

Supplement: Supplementary file 1 — Additional file 1: Supplementary Figure 1. ptPDX-derived CTCs are similar in morphology and traditionally defined CTC marker expression to patient CTCs. CTCs from matched patient and ptPDX blood were enriched on a microfilter and stained with CK 8/18/19-FITC, EpCAM-PE, CD45-Cy5 and DAPI for nuclei identification. Arrowhead showing a white blood cell (WBC) captured with CTC. Scale bar, 10 μm. Supplementary Figure 2. Histopathology and immunohistochemistry staining of patient-matched primary tumor, ptPDX and CDX tumor tissues. Immunohistochemical staining of patient-matched primary, ptPDX and CDX tumor tissues. A: Representative images of hematoxylin and eosin (H&E) stained and immunostained (CK7 and Napsin A) patient-matched tumor tissues from MU150. B: Representative images of H&E stained and immunostained (CK5/6 and p40) patient-matched tumor tissues from MU197. For both sets of staining, human aorta served as negative control tissue and IgG served as isotype control. Scale bar, 20 μm. Supplementary Figure 3. Bright field images of extracted nuclei suspension from automated cell counter after staining with trypan blue. Snap-frozen matched ptPDX and CDX tumor tissues were minced on ice and homogenized. Lysate was transferred through 70 μm cell strainer, homogenized again a few strokes, and passed through 40 μm cell strainer. Nuclei were counted after staining with Trypan blue using Countess II FL Automated Cell Counter to check the quality. Supplementary Figure 4. Pre-processing and filtering of snRNA-seq data matrix. Cells were filtered based on RNA transcript count, percentage of mitochondrial (mt)/ribosomal (rb) genes, percentage of mouse reads mapped in alignment to combined human-mouse reference genome. Red dotted line indicates the set cut-off applied to remove outliers. Supplementary Figure 5. Heatmaps using differentially expressed genes (DEGs) of all clusters of ptPDX and CDX tumor tissues for cell type annotation. DEGs (Supplementary file 1) having p adj o [file 12943_2022_1553_MOESM1_ESM.zip › 12943_2022_1553_MOESM1_ESM/Supplementary Figure 7.jpg]

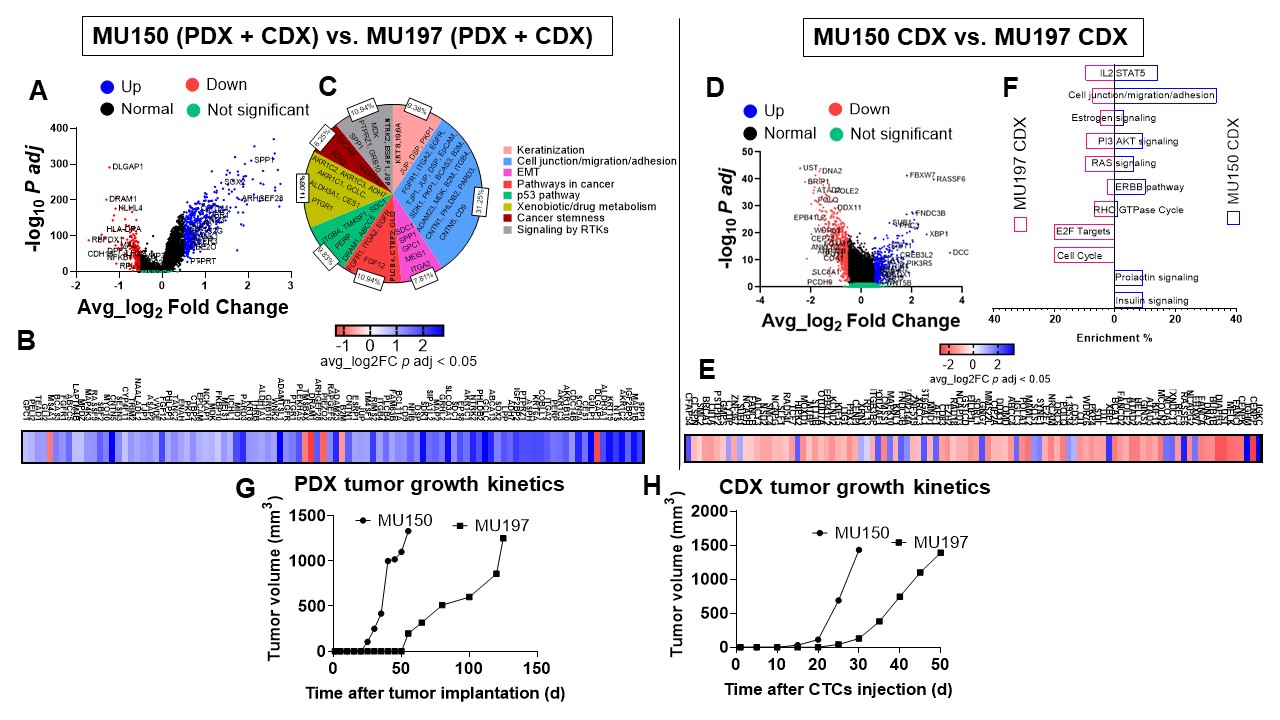

Supplement: Supplementary file 1 — Additional file 1: Supplementary Figure 1. ptPDX-derived CTCs are similar in morphology and traditionally defined CTC marker expression to patient CTCs. CTCs from matched patient and ptPDX blood were enriched on a microfilter and stained with CK 8/18/19-FITC, EpCAM-PE, CD45-Cy5 and DAPI for nuclei identification. Arrowhead showing a white blood cell (WBC) captured with CTC. Scale bar, 10 μm. Supplementary Figure 2. Histopathology and immunohistochemistry staining of patient-matched primary tumor, ptPDX and CDX tumor tissues. Immunohistochemical staining of patient-matched primary, ptPDX and CDX tumor tissues. A: Representative images of hematoxylin and eosin (H&E) stained and immunostained (CK7 and Napsin A) patient-matched tumor tissues from MU150. B: Representative images of H&E stained and immunostained (CK5/6 and p40) patient-matched tumor tissues from MU197. For both sets of staining, human aorta served as negative control tissue and IgG served as isotype control. Scale bar, 20 μm. Supplementary Figure 3. Bright field images of extracted nuclei suspension from automated cell counter after staining with trypan blue. Snap-frozen matched ptPDX and CDX tumor tissues were minced on ice and homogenized. Lysate was transferred through 70 μm cell strainer, homogenized again a few strokes, and passed through 40 μm cell strainer. Nuclei were counted after staining with Trypan blue using Countess II FL Automated Cell Counter to check the quality. Supplementary Figure 4. Pre-processing and filtering of snRNA-seq data matrix. Cells were filtered based on RNA transcript count, percentage of mitochondrial (mt)/ribosomal (rb) genes, percentage of mouse reads mapped in alignment to combined human-mouse reference genome. Red dotted line indicates the set cut-off applied to remove outliers. Supplementary Figure 5. Heatmaps using differentially expressed genes (DEGs) of all clusters of ptPDX and CDX tumor tissues for cell type annotation. DEGs (Supplementary file 1) having p adj o [file 12943_2022_1553_MOESM1_ESM.zip › 12943_2022_1553_MOESM1_ESM/Supplementary Figure 8.jpg]

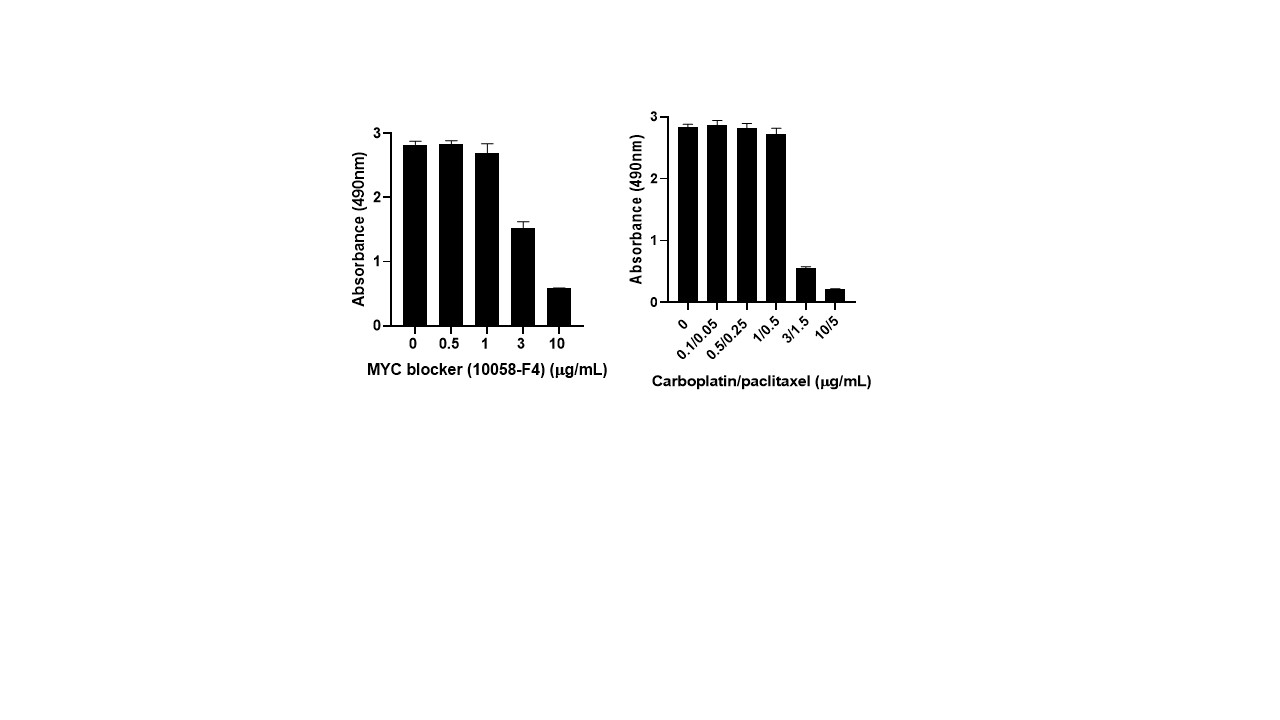

Supplement: Supplementary file 1 — Additional file 1: Supplementary Figure 1. ptPDX-derived CTCs are similar in morphology and traditionally defined CTC marker expression to patient CTCs. CTCs from matched patient and ptPDX blood were enriched on a microfilter and stained with CK 8/18/19-FITC, EpCAM-PE, CD45-Cy5 and DAPI for nuclei identification. Arrowhead showing a white blood cell (WBC) captured with CTC. Scale bar, 10 μm. Supplementary Figure 2. Histopathology and immunohistochemistry staining of patient-matched primary tumor, ptPDX and CDX tumor tissues. Immunohistochemical staining of patient-matched primary, ptPDX and CDX tumor tissues. A: Representative images of hematoxylin and eosin (H&E) stained and immunostained (CK7 and Napsin A) patient-matched tumor tissues from MU150. B: Representative images of H&E stained and immunostained (CK5/6 and p40) patient-matched tumor tissues from MU197. For both sets of staining, human aorta served as negative control tissue and IgG served as isotype control. Scale bar, 20 μm. Supplementary Figure 3. Bright field images of extracted nuclei suspension from automated cell counter after staining with trypan blue. Snap-frozen matched ptPDX and CDX tumor tissues were minced on ice and homogenized. Lysate was transferred through 70 μm cell strainer, homogenized again a few strokes, and passed through 40 μm cell strainer. Nuclei were counted after staining with Trypan blue using Countess II FL Automated Cell Counter to check the quality. Supplementary Figure 4. Pre-processing and filtering of snRNA-seq data matrix. Cells were filtered based on RNA transcript count, percentage of mitochondrial (mt)/ribosomal (rb) genes, percentage of mouse reads mapped in alignment to combined human-mouse reference genome. Red dotted line indicates the set cut-off applied to remove outliers. Supplementary Figure 5. Heatmaps using differentially expressed genes (DEGs) of all clusters of ptPDX and CDX tumor tissues for cell type annotation. DEGs (Supplementary file 1) having p adj o [file 12943_2022_1553_MOESM1_ESM.zip › 12943_2022_1553_MOESM1_ESM/Supplementary Figure 9.jpg]
