# Supplementary material for: Tumorigenic circulating tumor cells from xenograft mouse models of non-metastatic NSCLC patients reveal distinct single cell heterogeneity and drug responses
Source: Mol Cancer. 2022 Mar 12;21:73. doi: 10.1186/s12943-022-01553-5 (PMC8917773; doi:10.1186/s12943-022-01553-5)
Supplement: Supplementary file 3 — Additional file 3: Supplementary Table 1. Clinicopathological information and survival status of the NSCLC patients enrolled for ptPDX (N = 10) and CDX (N = 2) model development. Supplementary Table 2. Total counts of ptPDX individual CTCs and CTC clusters that were detected at the time of injection to naïve NSG mice to develop CDX models. [file 12943_2022_1553_MOESM3_ESM.zip › Supplementary Table 1.docx]

**Supplementary Table 1**. Clinicopathological information and survival status of the NSCLC patients enrolled for ptPDX (N=10) and CDX (N=2) model development.

| ***Patient ID*** | ***Age/***  ***gender*** | ***Histology (grade)*** | ***AJCC stage***  ***(TNM staging)*** | ***Treatment*** | ***Cancer recurrence***  ***(months)*** | ***Survival status***  ***(months)*** | ***ptPDX model established***  ***(months)*** | ***CDX model established***  ***(days)*** |
| --- | --- | --- | --- | --- | --- | --- | --- | --- |
| **MU150** | **68/male** | **AC**  **(G2)** | **IA1 (pT1aN0M0)** | **Surgery** | **No** | **Alive**  **(41)** | **Yes**  **(2)** | **Yes**  **(26)** |
| **MU179** | **65/female** | **AC**  **(G3)** | **IIB**  **(pT3N0M0)** | **Surgery + adjuvant chemotherapy** | **Yes (3)** | **Alive**  **(38)** | **Yes**  **(3)** | **No** |
| **MU189** | **74/male** | **SCC**  **(G2)** | **IIA (pT2bN0M0)** | **Surgery + adjuvant chemotherapy** | **No** | **Alive**  **(30)** | **Yes**  **(2)** | **No** |
| **MU190** | **85/male** | **SCC**  **(G2)** | **IA2 (pT1bN0M0)** | **Surgery** | **No** | **Alive**  **(29)** | **Yes**  **(8)** | **No** |
| **MU191** | **79/male** | **AC**  **(G2)** | **IA3 (pT1cN0M0)** | **Surgery** | **No** | **Deceased**  **(34)** | **Yes**  **(8)** | **No** |
| **MU197** | **56/male** | **SCC**  **(G2)** | **IIIA**  **(pT4N1M0)** | **Surgery + adjuvant chemotherapy**  **(cisplatin/doxetaxel)** | **No** | **Alive**  **(34)** | **Yes**  **(4)** | **Yes**  **(47)** |
| **MU233** | **66/male** | **SCC**  **(G2)** | **IA3 (pT1cN0M0)** | **Surgery** | **No** | **Deceased**  **(2)** | **Yes**  **(4)** | **No** |
| **MU236** | **72/female** | **AC**  **(G1)** | **IA2 (pT1bN0M0)** | **Surgery** | **No** | **Alive**  **(23)** | **Yes**  **(3)** | **No** |
| **MU258** | **52/female** | **LCNEC**  **(G3)** | **IIB**  **(pT3N0M0)** | **Surgery + adjuvant chemotherapy** | **No** | **Alive**  **(21)** | **Yes**  **(4)** | **No** |
| **MU279** | **59/male** | **AC**  **(G3)** | **IIA**  **(pT3N0M0)** | **Surgery** | **No** | **Alive**  **(18)** | **Yes**  **(3)** | **No** |

**Abbreviations**: AC: adenocarcinoma, SCC: squamous cell carcinoma, LCNEC: Large cell neuroendocrine carcinoma, G: histologic grading, AJCC: American Joint Committee on Cancer, TNM: Tumor, nodes, metastases, ptPDX: primary tumor patient-derived xenograft and CDX: circulating tumor cell-derived xenograft.
