# Supplementary material for: Tumorigenic circulating tumor cells from xenograft mouse models of non-metastatic NSCLC patients reveal distinct single cell heterogeneity and drug responses
Source: Mol Cancer. 2022 Mar 12;21:73. doi: 10.1186/s12943-022-01553-5 (PMC8917773; doi:10.1186/s12943-022-01553-5)
Supplement: Supplementary file 3 — Additional file 3: Supplementary Table 1. Clinicopathological information and survival status of the NSCLC patients enrolled for ptPDX (N = 10) and CDX (N = 2) model development. Supplementary Table 2. Total counts of ptPDX individual CTCs and CTC clusters that were detected at the time of injection to naïve NSG mice to develop CDX models. [file 12943_2022_1553_MOESM3_ESM.zip › Supplementary Table 2.docx]

**Supplementary Table 2**. Total counts of ptPDX individual CTCs and CTC clusters that were detected at the time of injection to naïve NSG mice to develop CDX models.

| **ptPDX**  **patient ID** | **Number of CTCs and CTC clusters detected in ptPDX blood**  **at the time of injection** | | **CDX growth** |
| --- | --- | --- | --- |
|  | **Individual CTCs** | **CTC clusters** |  |
| **MU150** | 620 | 93 | Yes |
| **MU179** | 545 | 11 | No |
| **MU189** | 20 | 7 | No |
| **MU190** | 601 | 29 | No |
| **MU191** | 235 | 6 | No |
| **MU197** | 600 | 89 | Yes |
| **MU233** | 300 | 8 | No |
| **MU236** | 25 | 5 | No |
| **MU258** | 150 | 7 | No |
| **MU279** | 505 | 10 | No |
