# Supplementary material for: Tumorigenic circulating tumor cells from xenograft mouse models of non-metastatic NSCLC patients reveal distinct single cell heterogeneity and drug responses
Source: Mol Cancer. 2022 Mar 12;21:73. doi: 10.1186/s12943-022-01553-5 (PMC8917773; doi:10.1186/s12943-022-01553-5)
Supplement: Supplementary file 4 — Additional file 4. Supplementary methods. [file 12943_2022_1553_MOESM4_ESM.docx]

**METHODS**

***NSCLC patient’s enrollment and sample collection***

Non-metastatic (as determined by standard staging with PET/CT, contrast-enhanced chest CT and brain MRI) NSCLC patients undergoing surgery were recruited between July 2018 to June 2020 and gave written informed consent (Table S1). Clinical data were prospectively collected by reviewing electronic medical records. The tumor staging manual of the American Joint Committee on Cancer (AJCC; 8^th^ edition) was applied [1]. Primary tumor tissues were collected at the time of lung resection.

***CTC detection from patient blood***

Blood draws from all the recruited patients were done before surgery to avoid cancer cell detachment by tissue manipulation, as described [2, 3]. First 6 mL of blood was discarded to avoid epithelial contamination from skin puncture and then, 7.5 mL of blood was collected in CellSave tubes (Menarini-Silicon Biosystems). Within 1 h, 7.5 mL of patient blood was diluted with equal volume of CellSieve^TM^ prefixation buffer and passed through a size-based microfilter with a pore size of 7 µm (CellSieve^TM^, Creatv MicroTech) using a syringe pump at a flow rate of 5 mL/min. CTCs trapped on the filter were then fixed with CellSieve^TM^ post-fixation buffer, permeabilized and characterized by immunofluorescence staining on the filter with an antibody cocktail (cytokeratin (CK) 8/18/19-FITC, Epithelial Cell Adhesion Molecule (EpCAM)-PE, CD45-Cy5) (CellSieve™ Enumeration Kit; Creatv MicroTech), and mounted using anti-fade mounting medium with 4′,6-diamidino-2-phenylindole (DAPI) (Cell Signaling Technology) [2-4] (Fig. S1). CTCs were identified based on the FDA-approved CellSearch® definition that a CTC is a CK/EpCAM+, CD45- cell with an intact DAPI+ nucleus [5].

***Development of NSCLC ptPDX models***

The immunodeficient mouse strain - NOD.Cg-*Prkdc^scid^* *Il2rg^tm1Wjl^*/SzJ (NOD scid gamma; NSG™, Jackson Laboratories) (aged 8-10 weeks; both genders in equal distribution) was used for xenografting. ptPDX models were developed by subcutaneous (s.c.) implantation of 1-3 fragments (~1.5 mm^3^) of the patient’s surgically resected lung tumor with 30% Matrigel (Corning). Tumor growths were determined by bi-weekly clinical volume measurements, and once tumors reached a size of ~1,000 mm^3^ mice were anaesthetized (100 mg/kg ketamine and 10 mg/kg xylazine) and blood was collected into Heparin Minicollect tubes (Greiner BioOne) by cardiac puncture. Then, mice were euthanized by cervical dislocation. Upon autopsies, tumors and organs were snap frozen or fixed in 10% buffered formalin for histological studies. A total of 20 PDX models were developed out of 40 patients recruited with a success rate of 50%. To maintain successful PDX colonies, excised tumor fragments were re-implanted to naive NSG mice for up to six generations in a minimum of 2 mice per generation.

***CTC detection from ptPDX blood to compare phenotypic expression with patient CTCs***

To compare patient CTCs with ptPDX-derived CTCs, 1 mL of blood from generation-2 ptPDX mice was diluted with equal volume of CellSieve^TM^ prefixation buffer and passed through a microfilter with a pore size of 7 µm (CellSieve^TM^, Creatv MicroTech) for size-based CTC enrichment. Microfilters were then stained with same set of anti-human antibody cocktail (CK 8/18/19-FITC, EpCAM-PE, CD45-Cy5), including anti-fade mounting medium with DAPI (Cell Signaling Technology) (Fig. S1). CTCs were identified as CK/EpCAM+, CD45- with an intact DAPI positive nucleus.

***Development of CDX mouse models with ptPDX-derived CTCs***

ptPDX-CTCs were enriched from the blood of ptPDXs by density gradient centrifugation (OncoQuick^®^; Greiner BioOne). 0.7-1 mL of blood was diluted with 4 volumes of sterile phosphate buffered saline (PBS) and layered carefully onto upper compartment of pre-cooled (4°C) OncoQuick^®^ tube and centrifuged at 1600 *g* at 4°C for 20 min in a swing bucket rotor with slow acceleration and no brake. After centrifugation, upper phase having plasma was carefully discarded and the entire liquid volume above the porous barrier was harvested to minimize the loss of CTCs. Harvested CTC fraction was washed with 50 mL of PBS and to the pellet 1 mL of DMEM/F12 medium was added and mixed well. 100 µL of CTC-containing medium was diluted with equal volume of PBS and cytocentrifuged on a microscopic slide. Cytospun slides were fixed and permeabilized, followed by immunostaining with primary antibodies against human pan-CK (mouse, 4545, 1:100; Cell Signaling Technology), human-CD45 (rabbit, ab10559; 1:100, Abcam), mouse CD45 (rat, ab25386, 1:100; Abcam) to confirm human origin of CTCs. Secondary antibodies used were goat anti-mouse IgG Alexa Fluor 488, donkey anti-rabbit IgG Alexa Fluor 555, goat anti-rat IgG Alexa Fluor 647 (1:500; ThermoFisher Scientific). CK positive CTCs and CTC clusters were enumerated by performing whole slide imaging (Table S2). To generate CDX models, ptPDX-CTCs were needle-injected (s.c.) in flanks with 30% Matrigel in 200 µL of DMEM/F12 media into naïve NSG mice and tumor growth was determined, as outlined above. CDX colonies were maintained up to six generations as mentioned above.

***Immunohistochemical staining of tumor tissues***

Patient-matched primary, ptPDX and CDX tumors were paraffin-embedded and immunostained for diagnostic pathology NSCLC markers with antibodies against CK7 (mouse, M7018, 1:100; Dako), Napsin A (mouse, ab73021, 1:100; Abcam), CK5/6 (mouse, ACR 105, 1:100; Biocare Medical), and p40 (rabbit, ACI 3030, 1:100; Biocare Medical) (Fig. S2). Additionally, c-MYC (Rabbit, 5605, 1:100; CST), Telomerase reverse transcriptase (TERT) (Rabbit, ab183105, 1:200; Abcam), cleaved Poly (ADB-ribose) polymerase (cPARP) (Rabbit, 5625, 1:50; CST) and cleaved Caspase-3 (cCASP3) (Rabbit, 9664, 1:1000; CST) antibodies were used to stain tumor tissues in chemotherapy experiments. Tissue sections were processed by de-paraffinization, rehydration, heat mediated antigen-retrieval, permeabilization and blocking. Tissue sections were incubated with primary antibodies overnight at 4 °C. HRP-conjugated secondary antibodies were incubated for 1 h at room temperature. Staining was developed with DAB and counterstaining was done with Hematoxylin. All immunostainings were reviewed by a clinical pathologist.

***Droplet-based single-nuclear RNA sequencing (snRNA-seq)***

*Sample Processing and Nuclei extraction:* Snap-frozen matched ptPDX and CDX tumor tissues were minced on ice and homogenized using Tissue-Tearor (Biospec) in cold Tris-HCl buffer (lysis Buffer) pH 7.4 containing 0.1% Triton X-100 and 0.2 U/µL RNase inhibitor (Promega). Lysate was transferred through 70 µm cell strainer, homogenized again a few strokes, and passed through 40 µm cell strainer. Filtrate was centrifuged at 500 *g* for 5 min at 4 °C and resuspended the pellet in PBS with 0.4% bovine serum albumin (BSA) and 0.2 U/µL RNase inhibitor (wash buffer and storage buffer). This step was repeated twice to wash the nuclei and finally counted after staining with Trypan blue using Countess II FL Automated Cell Counter (ThermoFisher Scientific) (Fig. S3). Suspensions were also stained with DAPI to confirm adequate dissociation and quality of nuclei.

*Library preparation and snRNA-seq.* Single-nuclei 3’ RNA-seq (snRNA-seq) library preparation and sequencing services were provided by the MU DNA Core Facility. Extracted nuclei suspended in 0.4% BSA in PBS with 0.2 U/µL RNase inhibitor at a concentration of 800 nuclei/µL were used for a targeted capture recovery of 3,000 nuclei per sample using the 10x Genomics Chromium System. Following encapsulation in a droplet, cDNA and library generation was performed according to the manufacturer’s protocol using the Chromium Single Cell 3’ Library Gel Bead kit (v2). Sequencing was performed using an Illumina NovaSeq 6000 to an average of 50,000 reads per nuclei.

*Computational analysis: De-multiplexing, Pre-Processing, and filtering.* Cellranger mkfastq was used for demultiplexing of raw files generated by Illumina sequencers. Each sample was separately aligned to a combined human-mouse reference genome and to a human reference genome. From the count matrix, which is an output of alignment to a combined reference, we calculated a percentage of mouse reads for each cell and set a threshold to find out the cells with the majority of mouse reads. Then, cells that had a majority of mouse reads were deleted from the count matrix, which is an output of alignment to a human reference genome. All the downstream analyses were performed for each sample separately using Seurat V3 package [6]. Cells with less than 200 genes and more than 5% mitochondrial and 10% ribosomal protein coding genes were excluded from further downstream analysis (Fig. S4). Cell doublets or multiplets may exhibit an aberrantly high gene count, so that outlier cells with high numbers of unique features were removed.

*Dimensionality reduction and clustering.* Seurat global-scaling normalization method “LogNormalize” was used to normalize the gene expression measurements for each cell by the total expression, multiplying it by a scale factor (×10,000, by default), and log-transforms the result. Based on appropriate thresholds of the mean expression and dispersion, 2000 highly variable genes were used for principal component analysis (PCA). Prior to PCA, a linear transformation was applied to scale and center features adjusting expression of each gene to give a mean expression across cells to be zero. Cells in each sample were clustered using unsupervised graph-based clustering algorithm implemented in Seurat. With the function “FindClusters” on 6-11 principal components (PCs) and resolution of 0.5-0.9, each cluster was annotated depending on the number of cells. Decision on the number of significant PCs to use in further analysis was made using JackStraw and ElbowPot functions. Uniform Manifold Approximation and Projection (UMAP), non-linear dimensionality reduction technique was utilized for better visualization and interpretation of clustering.

*Differentially expressed genes and cell type annotation.* Differentially expressed genes (DEGs) in each cluster were identified based on Wilcoxon Rank Sum (WRS) test using function “FindAllMarkers” (supplementary file 1). Cell type annotation was done using DEGs expression of cell-specific canonical markers of epithelial, mesenchymal, endothelial, and immune cell populations (supplementary file 2). Canonical markers were selected from the Lung Gene Expression Analysis (LGEA) portal [7], The Human Protein Atlas [8], Cell marker database [9], and recent publications by Karlsson et al. [10] and Laughney et al. [8].

*Integration of MU150 and MU197 CDXs datasets to determine differential Hallmark MYC target expression.* To avoid batch effect during differential analysis of MYC target genes enrichment from MU150 CDX and MU197 CDX, Seurat integration method was used. Differential expression between MU150 CDX and MU197 CDX samples was calculated using Model-based Analysis of Single-cell Transcriptomics (MAST) algorithm provided within the FindMarkers function in Seurat V3 with default parameters [11]. From differentially expressed genes, we selected MYC target genes [12] with a *p*-value <0.05.

*Aggregation of snRNA-seq data to determine gene expression similarities and differences within and across patient-matched PDX/CDX models.* All 4 patient-derived samples (MU150 PDX, MU150 CDX, MU197 PDX, MU197 CDX) were aggregated using harmony R package [13]. To analyze similarities between PDX and CDX samples from the same patient and across patients, we performed differential analysis between samples of interest using FindMarkers function from Seurat R packages. Differential analysis was done using Wilcoxon rank sum test between two samples. Differentially expressed genes were assigned to upregulated (UP; average log_2_ Fold Change > 0.5, adjusted *p* value < 0.05), normal (- 0.5 > average log_2_ Fold Change < 0.5, adjusted *p* value < 0.05), downregulated (down; average log_2_ Fold Change < 0.5, adjusted *p* value < 0.05) and not significant (adjusted *p* value > 0.05) groups and presented as volcano plots. Top 100 DEGs were used to perform gene set enrichment analysis [12] and pathway enrichment analysis [14, 15].

***Validation of single cell sequencing findings using open-access dataset***

To validate the results obtained from snRNA-seq of ptPDX and CDX models in patient tumors, we analyzed an external scRNA-seq data set of human NSCLC primary tumors and metastases (N=8 primary NSCLC lung tumors, N=5 distant NSCLC metastases) [8]. Raw scRNA-seq data were downloaded (accession number GSE123904). Pre-processing and filtering were conducted as mentioned above (cells with less than 200 genes, and more than 10% mitochondrial genes were excluded from downstream analysis). Clustering and DEGs were obtained using the Seurat V3 package [6].

***Chemosensitivity testing of CDX models with paclitaxel/carboplatin***

Carboplatin (Teva) and paclitaxel (Hospira) were diluted in sterile PBS. To accomplish sufficient statistical power with a total of N=5 mice per treatment group (chemotherapy versus vehicle control) per CDX model (MU150 and MU197), tumor fragments (~1.5 mm^3^) were excised from one parental CDX tumor and separately re-implanted s.c. into five naïve NSG mice per treatment group in each CDX model. Once the tumors reached a minimum size of 200 mm^3^, mice were then treated with doublet chemotherapy (carboplatin (40 mg/kg) and paclitaxel (20 mg/kg) in 200 µL sterile PBS) or vehicle control (castor oil in 200 µL sterile PBS) by intraperitoneal (i.p.) injection [16]. Treatment responses were determined by measuring s.c. tumor sizes with the analytic personnel blinded to treatment arms. Once the group had a tumor grown to a size of ~1,000 mm^3^, all animals were euthanized, autopsies performed and tumors collected for further analyses, as described above.

***Chemosensitivity testing of MU150 CDX cell line in vitro and MYC blockade***

MU150 CDX tumor was minced and enzymatically digested with 0.3 mg/mL each of collagenase type IV (Gibco), DNase I (Sigma) and Hyaluronidase (MP Biomedicals) in DMEM medium without FBS to obtain single cells. Suspension of cells was strained through 70 µm cell strainer and the filtrates were centrifuged and seeded into tissue culture dish that were allowed to acclimate for 24 hours. Cells were then treated with carboplatin (1 µg/mL) and paclitaxel (0.5 µg/mL), with or without 1 µg/mL of MYC blocker 10058-F4 (Tocris). Concentrations of carboplatin, paclitaxel and 10058-F4 were selected after dose titrations against the respective cell line (Fig. S9). Cells in media without any chemotherapeutic drugs or MYC blocker served as control. Treatment was carried out in triplicates and every 24 h treatment was terminated, and cells were either processed for live/dead cell staining or lysed for western blotting.

*Live/Dead cell staining and quantification.* After termination of treatment every 24 hours, cells were washed three times with PBS. Live/Dead Cell Double Staining Kit (Sigma) was used according to the manufacturer’s protocol. Following staining, cells were observed under the microscope (4X magnification) for live (green) and dead (red) cells. Quantification was performed by hybrid cell count or extraction (Keyence) that outputs extraction results into numerical values. Multiple *t* test was applied, and the results were considered statistically significant only with a *p* < 0.05.

*Western Blotting Analysis.* Cell lysates and tumor tissue lysates were prepared using T-PER™ Tissue Protein Extraction Reagent (Thermo Fisher Scientific). After quantitating (Bicinchoninic acid (BCA) method), an equal amount of protein was loaded to perform Western blotting as previously described [17]. Antibodies used were c-MYC (Rabbit, 5605, 1:1000; CST), TERT (Rabbit, ab183105, 1:1000; Abcam) and β-actin (Mouse, 3700, 1:1000; CST).

*Cell proliferation assay*. MU150 CDX tumor-derived cells (0.01 X 10^6^) were seeded in 96 well cell culture dish and treated with carboplatin (1 µg/mL) and paclitaxel (0.5 µg/mL) with or without MYC blocker 10058-F4 (1 µg/mL) up to 4 days. On each day of treatment, cell proliferation was recorded by measuring absorbance at 490 nm after 4 hours of adding CellTiter 96® AQueous One Solution (Promega) according to manufacturer’s protocol.

***In vivo chemosensitivity testing of MU150 CDX model and MYC blockade***

To evaluate the role of MYC blockade on chemotherapy response, a total of N=4 mice per treatment group (chemotherapy alone/MYC blocker (10058-F4) alone/chemotherapy + MYC blocker versus vehicle control) were selected. CDX tumor fragments (~1.5 mm^3^) were excised from one parental CDX tumor and separately re-implanted s.c. into four naïve NSG mice per treatment group. Once the tumor in all the groups reached a minimum size of 200 mm^3^, mice were then treated with doublet chemotherapy (carboplatin (40 mg/kg) and paclitaxel (20 mg/kg)), MYC blocker 10058-F4 (20 mg/kg for five days), chemotherapy + MYC blocker, or vehicle only (control) via i. p. injection. Cytotoxic agents were diluted to required concentrations in 200 µL of sterile PBS and one-time injections were performed. 10058-F4 was initially dissolved in DMSO to a concentration of 50 mg/mL and further diluted to required concentration in 500 µL (final DMSO concentration 0.016%) of DMEM media that was injected a day after carboplatin and paclitaxel injection. For MYC blocker alone and chemotherapy + MYC blocker groups’ 10058-F4 was injected 5 times every alternate day. For every injection 10058-F4 was freshly prepared from stock solution. Vehicle control group received castor oil in PBS on day of carboplatin/paclitaxel injections and 0.016 % DMSO in DMEM media during MYC blocker injections. Treatment responses were determined by measuring s.c. tumor sizes with the analytic personnel blinded to treatment arms. Once the group had a tumor growing to a size of ~1,000 mm^3^, all animals were euthanized, autopsies performed and tumors collected for further analyses, as described above.

***Statistical analysis***

For snRNA-seq data analyses statistical tests used were Wilcoxon Rank Sum test to find all genes in a cluster. Multiple comparison test (Boniferroni correction method) was applied to get adjusted *p* value for differentially expressed genes. Additional statistical tests applied were non-parametric Student *t*-test and multiple *t*-tests. Significance statements refer to a *p/p_adj_* value of < 0.05. Statistical analyses were performed using Prism (v8.00) (GraphPad Software).

**REFERENCES SUPPLEMENTARY METHODS**

1. Amin MB, American Joint Committee on Cancer., American Cancer Society.: *AJCC cancer staging manual.* Eight edition / editor-in-chief, Mahul B. Amin, MD, FCAP ; editors, Stephen B. Edge, MD, FACS and 16 others ; Donna M. Gress, RHIT, CTR - Technical editor ; Laura R. Meyer, CAPM - Managing editor. edn. Chicago IL: American Joint Committee on Cancer, Springer; 2017.

2. Manjunath Y, Mitchem JB, Suvilesh KN, Avella DM, Kimchi ET, Staveley-O'Carroll KF, Deroche CB, Pantel K, Li G, Kaifi JT: **Circulating giant tumor-macrophage fusion cells are independent prognosticators in non-small cell lung cancer patients.** *J Thorac Oncol* 2020.

3. Manjunath Y, Upparahalli SV, Suvilesh KN, Avella DM, Kimchi ET, Staveley-O'Carroll KF, Li G, Kaifi JT: **Circulating tumor cell clusters are a potential biomarker for detection of non-small cell lung cancer.** *Lung Cancer* 2019, **134:**147-150.

4. Adams DL, Stefansson S, Haudenschild C, Martin SS, Charpentier M, Chumsri S, Cristofanilli M, Tang CM, Alpaugh RK: **Cytometric characterization of circulating tumor cells captured by microfiltration and their correlation to the CellSearch((R)) CTC test.** *Cytometry A* 2015, **87:**137-144.

5. Cristofanilli M, Budd GT, Ellis MJ, Stopeck A, Matera J, Miller MC, Reuben JM, Doyle GV, Allard WJ, Terstappen LW, Hayes DF: **Circulating tumor cells, disease progression, and survival in metastatic breast cancer.** *N Engl J Med* 2004, **351:**781-791.

6. Stuart T, Butler A, Hoffman P, Hafemeister C, Papalexi E, Mauck WM, 3rd, Hao Y, Stoeckius M, Smibert P, Satija R: **Comprehensive Integration of Single-Cell Data.** *Cell* 2019, **177:**1888-1902 e1821.

7. Du Y, Kitzmiller JA, Sridharan A, Perl AK, Bridges JP, Misra RS, Pryhuber GS, Mariani TJ, Bhattacharya S, Guo M, et al: **Lung Gene Expression Analysis (LGEA): an integrative web portal for comprehensive gene expression data analysis in lung development.** *Thorax* 2017, **72:**481-484.

8. Laughney AM, Hu J, Campbell NR, Bakhoum SF, Setty M, Lavallee VP, Xie Y, Masilionis I, Carr AJ, Kottapalli S, et al: **Regenerative lineages and immune-mediated pruning in lung cancer metastasis.** *Nat Med* 2020, **26:**259-269.

9. Zhang X, Lan Y, Xu J, Quan F, Zhao E, Deng C, Luo T, Xu L, Liao G, Yan M, et al: **CellMarker: a manually curated resource of cell markers in human and mouse.** *Nucleic Acids Res* 2019, **47:**D721-D728.

10. Karlsson M, Zhang C, Mear L, Zhong W, Digre A, Katona B, Sjostedt E, Butler L, Odeberg J, Dusart P, et al: **A single-cell type transcriptomics map of human tissues.** *Sci Adv* 2021, **7**.

11. Finak G, McDavid A, Yajima M, Deng J, Gersuk V, Shalek AK, Slichter CK, Miller HW, McElrath MJ, Prlic M, et al: **MAST: a flexible statistical framework for assessing transcriptional changes and characterizing heterogeneity in single-cell RNA sequencing data.** *Genome Biol* 2015, **16:**278.

12. Subramanian A, Tamayo P, Mootha VK, Mukherjee S, Ebert BL, Gillette MA, Paulovich A, Pomeroy SL, Golub TR, Lander ES, Mesirov JP: **Gene set enrichment analysis: a knowledge-based approach for interpreting genome-wide expression profiles.** *Proc Natl Acad Sci U S A* 2005, **102:**15545-15550.

13. Korsunsky I, Millard N, Fan J, Slowikowski K, Zhang F, Wei K, Baglaenko Y, Brenner M, Loh PR, Raychaudhuri S: **Fast, sensitive and accurate integration of single-cell data with Harmony.** *Nat Methods* 2019, **16:**1289-1296.

14. Huang da W, Sherman BT, Lempicki RA: **Systematic and integrative analysis of large gene lists using DAVID bioinformatics resources.** *Nat Protoc* 2009, **4:**44-57.

15. Huang da W, Sherman BT, Lempicki RA: **Bioinformatics enrichment tools: paths toward the comprehensive functional analysis of large gene lists.** *Nucleic Acids Res* 2009, **37:**1-13.

16. Jensen MM, Erichsen KD, Johnbeck CB, Bjorkling F, Madsen J, Jensen PB, Sehested M, Hojgaard L, Kjaer A: **[18F]FDG and [18F]FLT positron emission tomography imaging following treatment with belinostat in human ovary cancer xenografts in mice.** *BMC Cancer* 2013, **13:**168.

17. Liu X, Huang Y, Yuan H, Qi X, Manjunath Y, Avella D, Kaifi JT, Miao Y, Li M, Jiang K, Li G: **Disruption of oncogenic liver-intestine cadherin (CDH17) drives apoptotic pancreatic cancer death.** *Cancer Lett* 2019, **454:**204-214.
